# Optimized protoplast isolation and transfection with a breakpoint: accelerating Cas9/sgRNA cleavage efficiency validation in monocot and dicot

**DOI:** 10.1007/s42994-024-00139-7

**Published:** 2024-04-15

**Authors:** Debasmita Panda, Subhasis Karmakar, Manaswini Dash, Swagat Kumar Tripathy, Priya Das, Sagar Banerjee, Yiping Qi, Sanghamitra Samantaray, Pradipta Kumar Mohapatra, Mirza J. Baig, Kutubuddin A. Molla

**Affiliations:** 1 ICAR National Rice Research Institute, Cuttack, Odisha 753006 India; 2 Department of Botany, Ravenshaw University, Cuttack, Odisha 753003 India; 3 Department of Plant Science and Landscape Architecture, University of Maryland, College Park, MD 20742 USA

**Keywords:** Protoplast, Genome editing, Transgene-free edited plants, Rice, Chickpea

## Abstract

The CRISPR-Cas genome editing tools are revolutionizing agriculture and basic biology with their simplicity and precision ability to modify target genomic loci. Software-predicted guide RNAs (gRNAs) often fail to induce efficient cleavage at target loci. Many target loci are inaccessible due to complex chromatin structure. Currently, there is no suitable tool available to predict the architecture of genomic target sites and their accessibility. Hence, significant time and resources are spent on performing editing experiments with inefficient guides. Although in vitro-cleavage assay could provide a rough assessment of gRNA efficiency, it largely excludes the interference of native genomic context. Transient in-vivo testing gives a proper assessment of the cleavage ability of editing reagents in a native genomic context. Here, we developed a modified protocol that offers highly efficient protoplast isolation from rice, *Arabidopsis,* and chickpea, using a sucrose gradient, transfection using PEG (polyethylene glycol), and validation of single guide RNAs (sgRNAs) cleavage efficiency of CRISPR-Cas9. We have optimized various parameters for PEG-mediated protoplast transfection and achieved high transfection efficiency using our protocol in both monocots and dicots. We introduced plasmid vectors containing Cas9 and sgRNAs targeting genes in rice, *Arabidopsis,* and chickpea protoplasts. Using dual sgRNAs, our CRISPR-deletion strategy offers straightforward detection of genome editing success by simple agarose gel electrophoresis. Sanger sequencing of PCR products confirmed the editing efficiency of specific sgRNAs. Notably, we demonstrated that isolated protoplasts can be stored for up to 24/48 h with little loss of viability, allowing a pause between isolation and transfection. This high-efficiency protocol for protoplast isolation and transfection enables rapid (less than 7 days) validation of sgRNA cleavage efficiency before proceeding with stable transformation. The isolation and transfection method can also be utilized for rapid validation of editing strategies, evaluating diverse editing reagents, regenerating plants from transfected protoplasts, gene expression studies, protein localization and functional analysis, and other applications.

**Supplementary Information:**

The online version contains supplementary material available at 10.1007/s42994-024-00139-7.

## Introduction

Protoplasts, which are plant cells devoid of cell walls, have a unique capability to efficiently uptake various macromolecules (such as DNA, RNA, and proteins). This ability makes them highly suitable for transient gene expression analysis, providing valuable insights into gene expression dynamics and regulatory mechanisms in plant biology (Shen et al. 2017; Xu et al. 2020). In addition, protoplast transfection using polyethylene glycol (PEG) is a simple and cost-effective method for studying gene function, subcellular localization, and protein–protein interaction in various plant species (Yu et al. 2017; Priyadarshani et al. 2018). Although using protoplasts may seem like a method of the past, it witnessed a remarkable resurgence with the advent of gene editing technologies. Protoplasts are now used in a wide array of genome editing applications, including gRNA efficiency assessment (Li et al. 2013), transgene-free editing (Zhang et al. 2022), base editing (Molla et al. 2021; Xiong et al. 2023), prime editing (Perroud et al. 2023), and CRISPR activation (Li et al. 2017; Pan et al. 2021).

The CRISPR-Cas9 editing system consists of two components: the Cas9 protein and the single guide RNA (sgRNA). Cas9 is an RNA-guided DNA endonuclease, whereas sgRNA contains a 5′ 20 nucleotide protospacer sequence that is complementary to the target DNA sequence, directing Cas9 to the target site. Cas9 makes double-strand breaks (DSBs) in the target genomic loci. As a result of DSB repair, by cellular machinery, edits are generated in the form of random insertions and deletions (Indels). More than one genomic locus could be targeted by simply providing additional specific gRNAs. The sgRNA-Cas9 complex can be delivered into protoplasts as plasmids having sgRNA and coding sequence of Cas9 with suitable promoter (Metje-Sprink et al. 2019) or as ribonucleoprotein complexes (Svitashev et al. 2016; He and Zhao 2020). Protoplast studies offer a rapid and high-throughput method for evaluating gene expression and in vivo genome editing efficiency.

CRISPR-Cas system may fail to target genomic sites that are inaccessible due to tightly packed chromatin structure (Yarrington et al. 2018). If the RNP complex cannot reach the intended target sites, no sequence modification will occur (Brandt et al. 2020). One of the most critical factors driving the editing outcome is the sgRNA sequence that guides the Cas9 protein to cleave the target DNA site (Liu et al. 2022). Since stable transformation and plant regeneration are time-consuming, working with inefficient sgRNA can cause a significant loss of time. Therefore, researchers desire a rapid method to evaluate sgRNA efficiency before proceeding to stable transformation. Protoplast offers an excellent in vivo system to serve the above purpose. For the genome editing experiment, protoplasts were isolated from leaves or seedlings and utilized in diverse plant species (Malnoy et al. 2016; Zhang et al. 2016; Andersson et al. 2018; Murovec et al. 2018; Brandt et al. 2020; De Bruyn et al. 2020; Lee et al. 2020; Hsu et al. 2021). Nonetheless, the routine utilization of protoplasts across laboratories is impeded by challenges such as the low viability of isolated protoplasts and the limited efficiency of transfection. Moreover, a protocol with a breakpoint between isolation and transfection of protoplasts is highly desired by researchers.

In the present study, we have established a simplified protoplast isolation and transfection procedure in model monocot (rice) and dicots (*Arabidopsis*, and chickpea) with a pause point in-between. By varying the time for enzymatic digestion, and using sucrose gradient, we optimized conditions to obtain highly viable protoplasts suitable for downstream applications. We observed the age of the plant materials, protoplasts viability, and yields are crucial factors governing the transfection efficiency. We have validated CRISPR-Cas9 mutagenesis efficiency for different targets from rice, *Arabidopsis*, and chickpea, related to height, lodging resistance, yield, stress tolerance, amino acid biosynthesis, carotenoid biosynthesis, and protein metabolism, using the developed method within less than a week. By demonstrating this protoplast platform in three plant species, we present here a replicable method that will facilitate testing of nuclease variants, gRNAs, and different experimental designs, in diverse plant species.

## Results

### Establishment of a high-quality protoplast isolation protocol

**Fig. 1:**
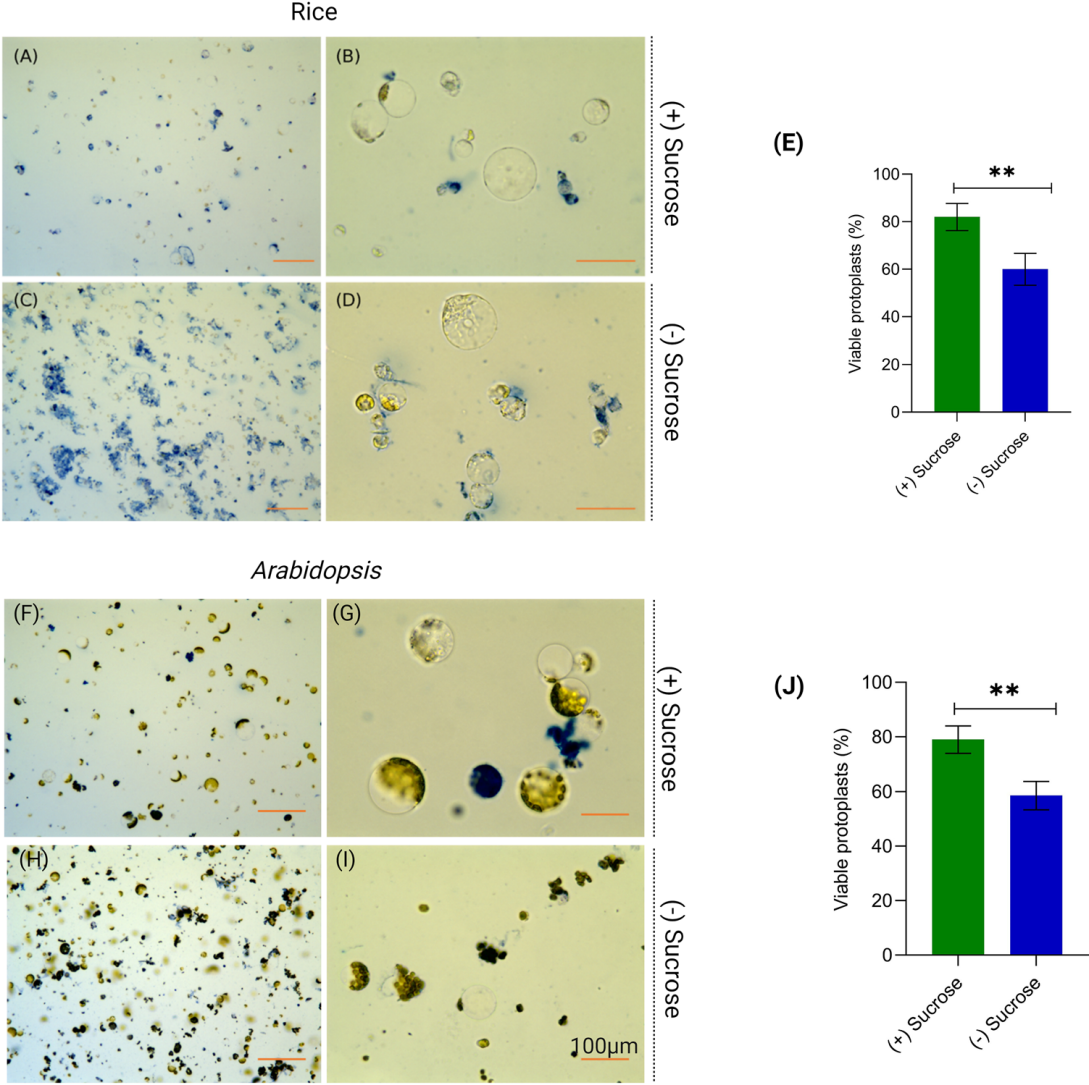
Viability of isolated protoplasts from rice and *Arabidopsis* assessed by Evan’s blue staining. **A**–**D** 10× and 40× view of Evan’s blue-stained isolated protoplasts from rice using sucrose gradient and without sucrose gradient. **E** Effects of (+) sucrose gradient and (-) sucrose gradient on viability of rice protoplasts. **F**–**I** 10× and 40× view of Evan’s blue-stained isolated protoplasts from *Arabidopsis* using sucrose gradient and without sucrose gradient. **J** Effects of (+) sucrose gradient and (-) sucrose gradient on viability of *Arabidopsis* protoplasts. Each bar represents mean ± SE for five replicates

The protoplast isolation method described here was repeated at least five times for both Indica and Japonica rice cultivars as well as for *Arabidopsis* and chickpea. Digestion for 5 h generates a protoplast yield averaging from 2 × 10^7^/mL to 5 × 10^7^/mL for all the plants used in the study. The final protoplast concentration was adjusted to 2 × 10^6^ cells/mL for use in transfection. Usually, a significant number of non-viable cells are present in the solution after the digestion. It is important to evaluate protoplast viability after each isolation procedure and separate viable cells from non-viable ones, which improves transfection and editing efficiency. Non-viable cells present false signals, hampering efficiency. We have standardised this protoplast isolation procedure using a sucrose gradient, which removes the broken and degraded protoplasts from the final collection, and increases the number of intact, large, and viable protoplasts (Supplementary Fig. S2). Evans Blue dye binds to cell membranes and can only penetrate cells with compromised or damaged cell membranes. Healthy, viable cells have intact cell membranes that prevent the dye from entering, while damaged or dead cells allow the dye to penetrate. Evans Blue staining of isolated protoplasts revealed that the inclusion of the sucrose gradient step has significantly improved the yield of viable protoplasts in both rice (*P* < 0.0001) and *Arabidopsis* (*P* < 0.0001) (Fig. 1). In the case of rice, the viability of protoplasts was observed to be 80%, when the sucrose gradient step was included, and 50% when it was omitted (Fig. 1A–E). For *Arabidopsis*, a similar trend was observed, with viability percentages of 76% and 50% in the presence and absence of the sucrose gradient step, respectively (Fig. 1F–J).

**Fig. 2:**
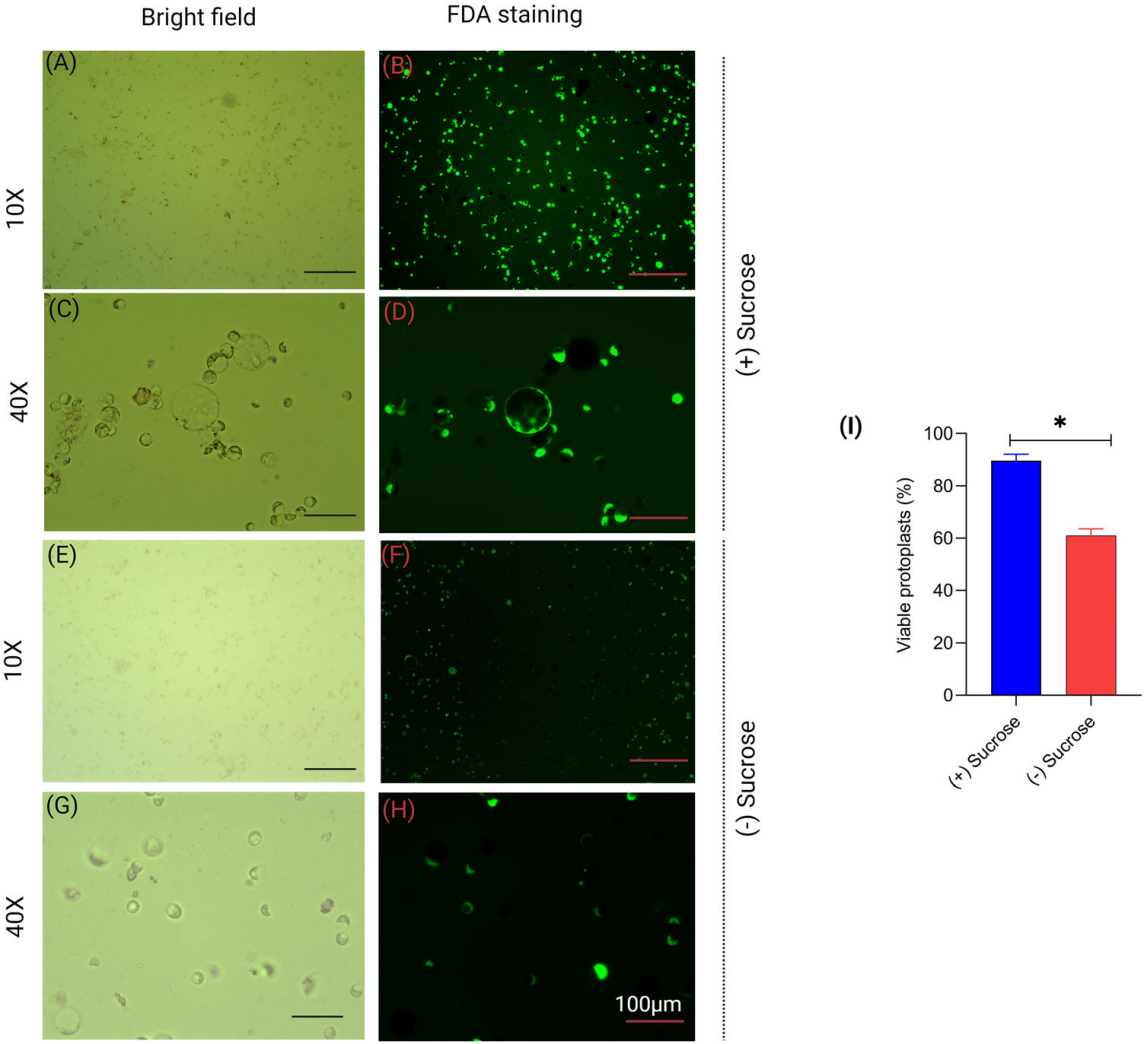
Viability of isolated protoplasts from rice tissues assessed with 0.01% fluorescein diacetate (FDA) staining. Viable protoplasts show fluorescence and non-viable remain dark. **A** and **C** Bright field view of FDA-stained isolated protoplasts from rice using sucrose gradient. **E** and **G** Bright field view of FDA-stained isolated protoplasts from rice without sucrose gradient. **B** and **D** Fluorescent protoplasts using sucrose gradient. **F** and **H** Fluorescent protoplasts without sucrose gradient. **I** Effects of (+) sucrose gradient and (-) sucrose gradient on the viability of rice protoplasts. Each bar represents mean ± SE for three replicates

To further confirm the effect of the sucrose gradient, rice protoplast viability was checked and evaluated with FDA. FDA is a non-fluorescent compound that can freely penetrate the plasma membrane of protoplasts. Once the FDA enters viable cells with intact membranes, it is hydrolyzed by intracellular esterase to produce fluorescein, a highly fluorescent compound. Since this conversion only occurs in live and metabolically active cells, FDA staining is reliably used to check viability. Our staining data showed a yield of 91% viable protoplasts with the sucrose gradient step, while it was 60% without the step (Fig. 2A–I).

**Fig. 3:**
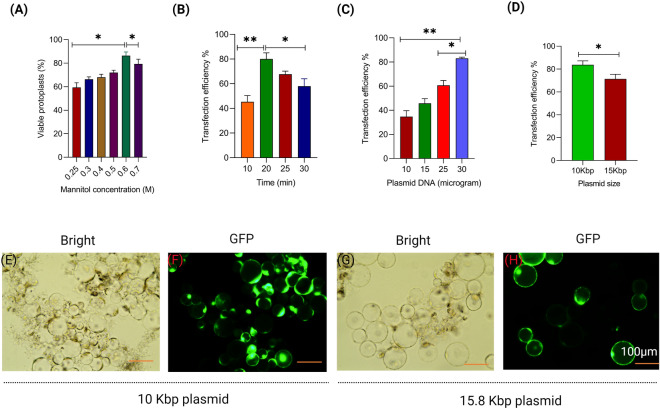
Optimization of transient transfection system in rice protoplasts. **A** Bar graph showing effects of different concentrations of D-mannitol on protoplast viability. **B** Bar graph showing effects of incubation time with PEG-CaCl_2_ on protoplasts transfection efficiency. **C** Effects of plasmid DNA concentration on transient transfection. **D** Effects of plasmid size on protoplasts transfection. **E** and **F** Images of protoplast (transfected with smaller size plasmid DNA) viewed under bright field and GFP filter; **G** and **H** Images of protoplasts (transfected with larger size plasmid DNA) viewed under bright field and GFP filter. Each bar represents mean ± SE for three replicates

The duration of cell wall digestion is a crucial factor affecting both the yield and viability of the protoplasts. We tested various digestion times for rice seedlings and determined that a 5-h incubation period produced the highest number of viable protoplasts, with no further improvement beyond that time. Mannitol plays a vital role in ensuring the successful isolation of protoplasts by maintaining their structural and functional integrity through establishing osmotic balance, preventing damage, and facilitating subsequent experiments and applications involving protoplasts. We used different concentrations of mannitol (0.3, 0.4, 0.5, and 0.6 M) in the initial solution to incubate cut strips and in the protoplast isolation buffer. We observed that a 0.6 M mannitol solution yielded the highest protoplast count with viability exceeding 85% for rice (Fig. 3A).

### Optimization of PEG-mediated protoplast transfection

To evaluate the suitability of the isolated protoplasts for transient gene expression analysis, we have performed PEG-Ca^2+^ mediated transfection of reporter plasmids. The vectors pRGE-GFP and pRGE-BFP were used to calculate the protoplast transfection efficiency for rice and determine the optimal incubation time and plasmid DNA concentration. The highest efficiency was observed when protoplasts were incubated for 20 min (Fig. 3B). It was observed that the concentration of plasmid DNA had a major influence on the transfection efficiency. Using purified plasmid DNA concentration from 10 to 30 µg in rice increases the transfection efficiency from 55 to 80% in rice (Fig. 3C). Additionally, we have transfected two different sizes (10 kb and 15 kb) of the plasmids harboring the same GFP expression cassette (*Ubi::GFP*). As shown in Fig. 3D, smaller plasmid had an advantage over larger ones in obtaining higher transfection efficiency. A transfection efficiency of approximately 65% was observed using the pRGEB-GFP vector (~ 15 kb), while an efficiency of over 80% was achieved with the smaller size plasmid, pRGE-GFP (~ 10 kb) (Fig. 3D, E–H).

We also checked the plasmid DNA concentration for *Arabidopsis* and chickpea and established that 45 µg of plasmid instead of 30 µg improved transfection efficiency. For *Arabidopsis*, we have used two vectors with two different promoters controlling the expression of GFP. GFP was found to be expressed higher with the eCAMV35S promoter than with the AtUbi10 promoter (Supplementary Fig. S3). For Chickpea, we have used eCaMV: GFP for evaluating transfection efficiency. Ultimately, the optimized conditions for PEG-CaCl_2_-mediated protoplast transfection in rice were as follows: 10 days old dark-grown etiolated rice seedlings, digestion time 5 h, plasmid DNA of 30 µg, and a 100 mM CaCl_2_ concentration, with an incubation time of 20 min. The optimized conditions for *Arabidopsis* and chickpea were as follows: 10–12 (size > 1 cm) leaves of 2–3 weeks old *Arabidopsis* or chickpea plants, digestion time of 5 h, plasmid DNA of 45 µg, and 100 mM CaCl_2_ concentration, with an incubation of 20 min.

### Sucrose gradient increases yield, viability, and transfection efficiency of protoplasts

**Fig. 4:**
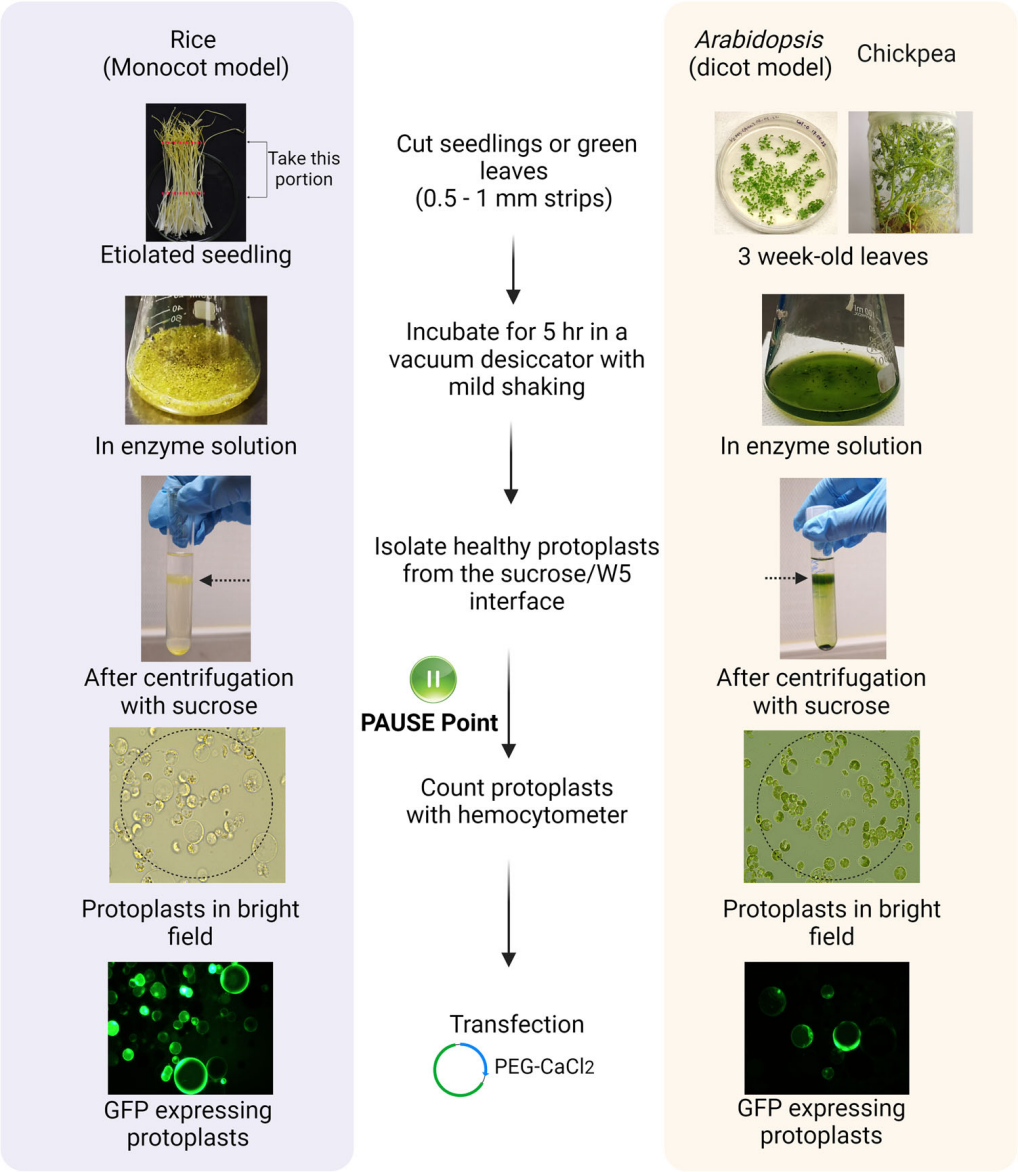
Schematic representation of protoplast isolation from etiolated rice seedlings and green leaves of *Arabidopsis* and chickpea. After centrifuging with 0.55 M sucrose, protoplast collected from the interface can be stored at 25 °C in the dark for 24/48 h with little loss of viability

**Fig. 5:**
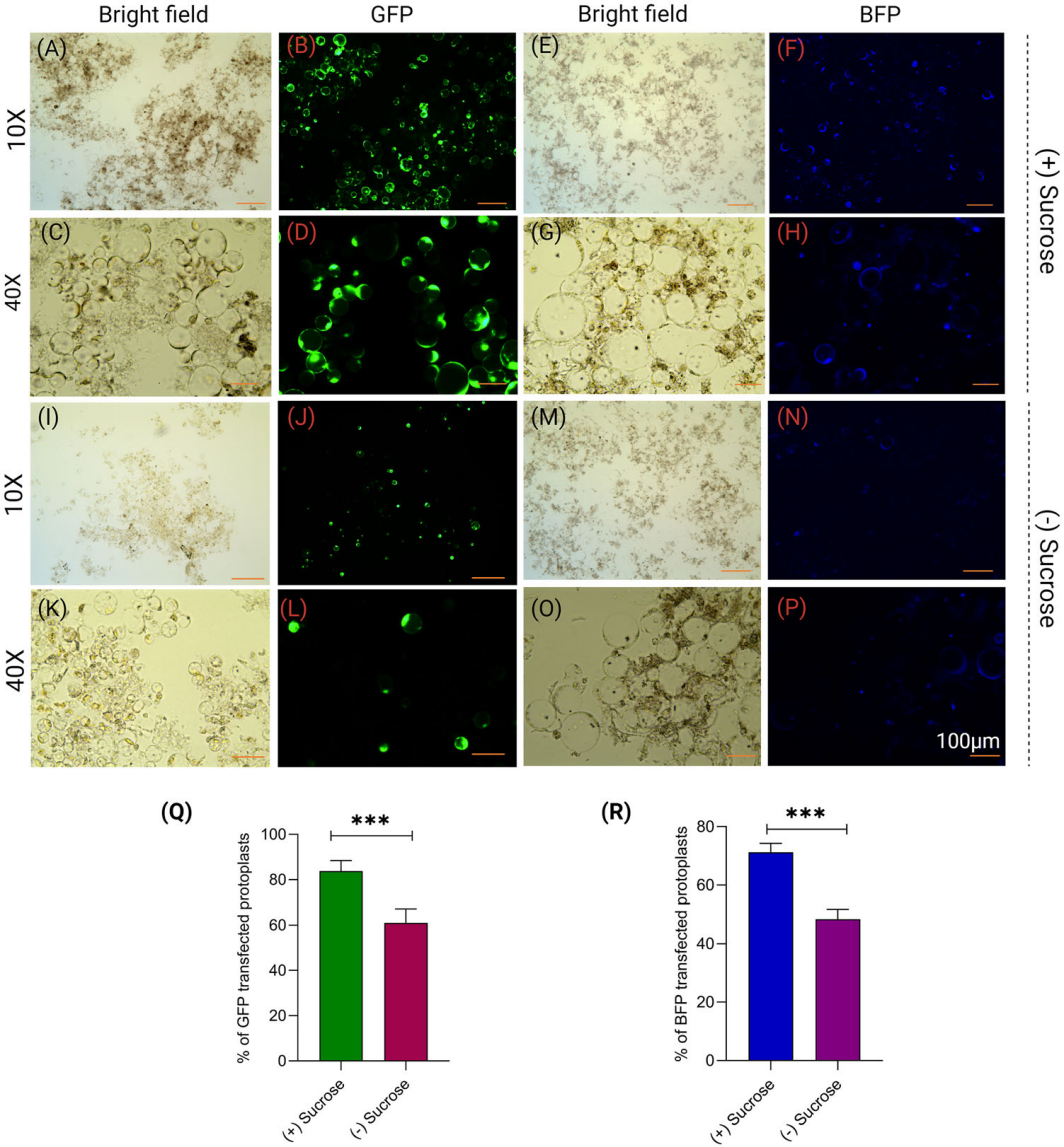
Effect of sucrose gradient on transfection efficiency. **A–D** Bright field and GFP filter image of protoplasts isolated with (+) sucrose gradient. **E–H** Bright field and BFP filter image of protoplasts isolated with (+) sucrose gradient. **I–L** Bright field and GFP filter image of protoplasts isolated without (-) sucrose gradient. **M–P** Bright field and BFP filter image of protoplasts isolated without (-) sucrose gradient. **Q** Bar graph showing the percentage of GFP transfected protoplast isolated using (+) and (-) sucrose gradient. **R** Bar graph showing the percentage of BFP transfected protoplast isolated using (+) and (-) sucrose gradient. Each bar represents mean ± SE for five replicates

After digestion in the enzymatic solution, the protoplast solution was filtered and centrifuged using a 0.55 M sucrose solution. Adding a gentle overlay of W5 solution over the sucrose layer was found to be a crucial step for achieving optimal protoplast yield. Following centrifugation with the 0.55 M sucrose, healthy and large protoplasts accumulated at the W5-sucrose interface (Fig. 4). We also conducted a separate set of protoplast isolations without using the 0.55 M sucrose solution, which failed to effectively separate the protoplasts from debris (Fig. 1). The band of protoplasts near the W5-sucrose interface was extracted using 200 µL pipettes with cut tips. Finally, the protoplasts were washed in W5 solution with low-speed centrifugation to increase purity. Significant variations were observed in the transfection efficiency and viability of protoplasts isolated with and without the sucrose gradient. The percentage of non-viable protoplasts increased significantly (nearly 65%) when the sucrose gradient was not used, while the use of sucrose resulted in a large number of intact viable protoplasts (nearly 85%) (Fig. 1). The transfection efficiency exhibited greater variation for both pRGE-GFP and pRGE-BFP when using sucrose (> 80%) as compared to without sucrose (~ 50%) (Fig. 5A–R). Taken together, these results suggest that employing the sucrose step in the isolation protocol significantly enhances the concentration of healthy protoplasts and, thereby, improves transfection efficiency to a great extent. All the troubleshooting is listed in the supplementary file.

### Including a breakpoint by protoplasts preservation and impact on transfection efficiency

Based on current literature and our knowledge, protoplast isolation is considered as a complex task, requiring substantial time and labour. Additionally, the storage of protoplasts highlights significant challenges due to the risk of cell rupture. Prolonged isolation times, coupled with multiple sets of transfections, often necessitate researchers to remain in the lab for nearly 12–15 h in a single working day. It would be more convenient if the protocol allowed the option to postpone the transfection step to the next working day by preserving isolated protoplasts without compromising viability. In this regard, we investigated the possibility of preserving isolated protoplasts for 24 and 48 h.

**Fig. 6:**
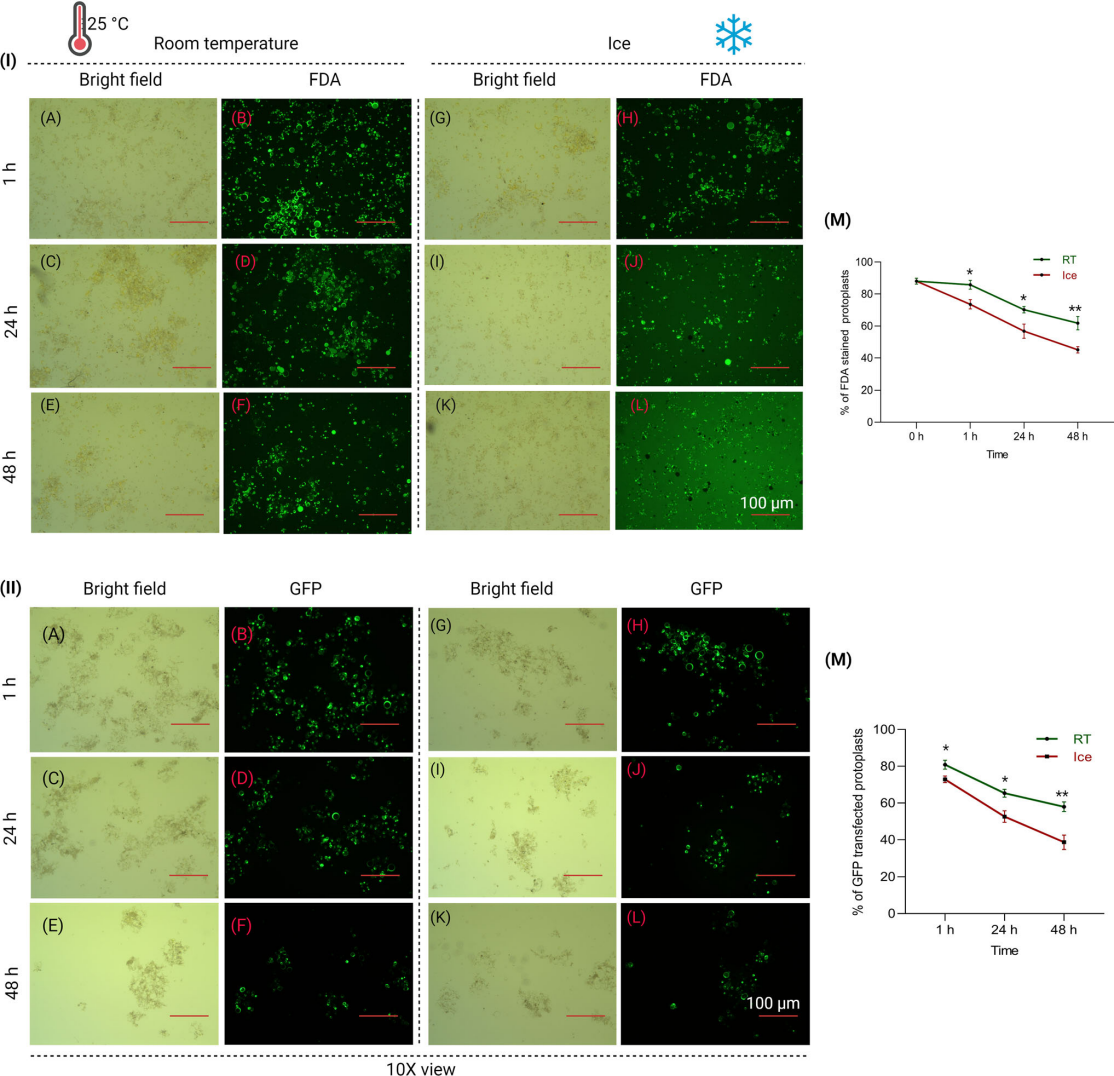
Effects of protoplast storage on viability and transfection efficiency. **I-A** to **I-L** Images depict the viability of isolated protoplasts at different time intervals (1 h, 24 h, and 48 h) at room temperature (25 °C) and on ice as visualized with FDA staining. **I-M** Graph depicting viability of protoplast. **II-A** to **II-L** *Ubi::GFP* transfection efficiency of isolated protoplasts at different storage time intervals (1 h, 24 h, and 48 h) at room temperature and on ice. **II-M** Graph illustrating the percentage of GFP-transfected protoplasts at different storage time intervals and temperatures. Each data point represents the mean ± SE for three replicates. (*) depicts statistical significance at *P* < 0.05; (**) depicts statistical significance at *P* < 0.01

An equal number of protoplasts collected from the sucrose gradient were stored in MMG solution at room temperature (RT) (25 °C) and on ice for up to 48 h. Isolated protoplasts survived for two days at RT (25 °C) with a slight decrease in viable protoplast concentration (70% and 61% at 24 and 48 h, respectively, versus 86% at 1 h) (Fig. 6I-A to 6I-F and 6I-M). In comparison to room temperature-stored protoplasts, we observed a significant (*P* < 0.05) decrease in viability (57% and 45% at 24 and 48 h, respectively, versus 74% at 1 h) when protoplasts were stored on ice (Fig. 6I-G to I-L and I-M). Bright-field observation of protoplasts revealed more pronounced rupture and aggregation when stored on ice compared to storage at room temperature (RT) (Supplementary Fig. S4).

We performed *OsUbi::GFP* transfection on the stored rice protoplasts at two different storage conditions after 1 h, 24 h and 48 h and evaluated transfection efficiency through GFP expression. The transfection efficiency of protoplasts was the highest (80%) at 1 h of RT storage, followed by 65% and 57% at 24 and 48 h, respectively (Fig. 6II-A to II-F and II-M). Compared to RT-stored protoplast, we observed that GFP transfection efficiency significantly decreased (*P* < 0.05) in ice-stored protoplasts, with values of 72% at 1 h, followed by 52% and 38% at 24 and 48 h, respectively (Fig. 6II-G to II-L and II-M). The results indicate that we have the flexibility to pause the transfection experiment for a day or two with RT-stored protoplasts.

### Rapid validation of CRISPR-Cas9 constructs for genome editing in isolated protoplasts

**Fig. 7:**
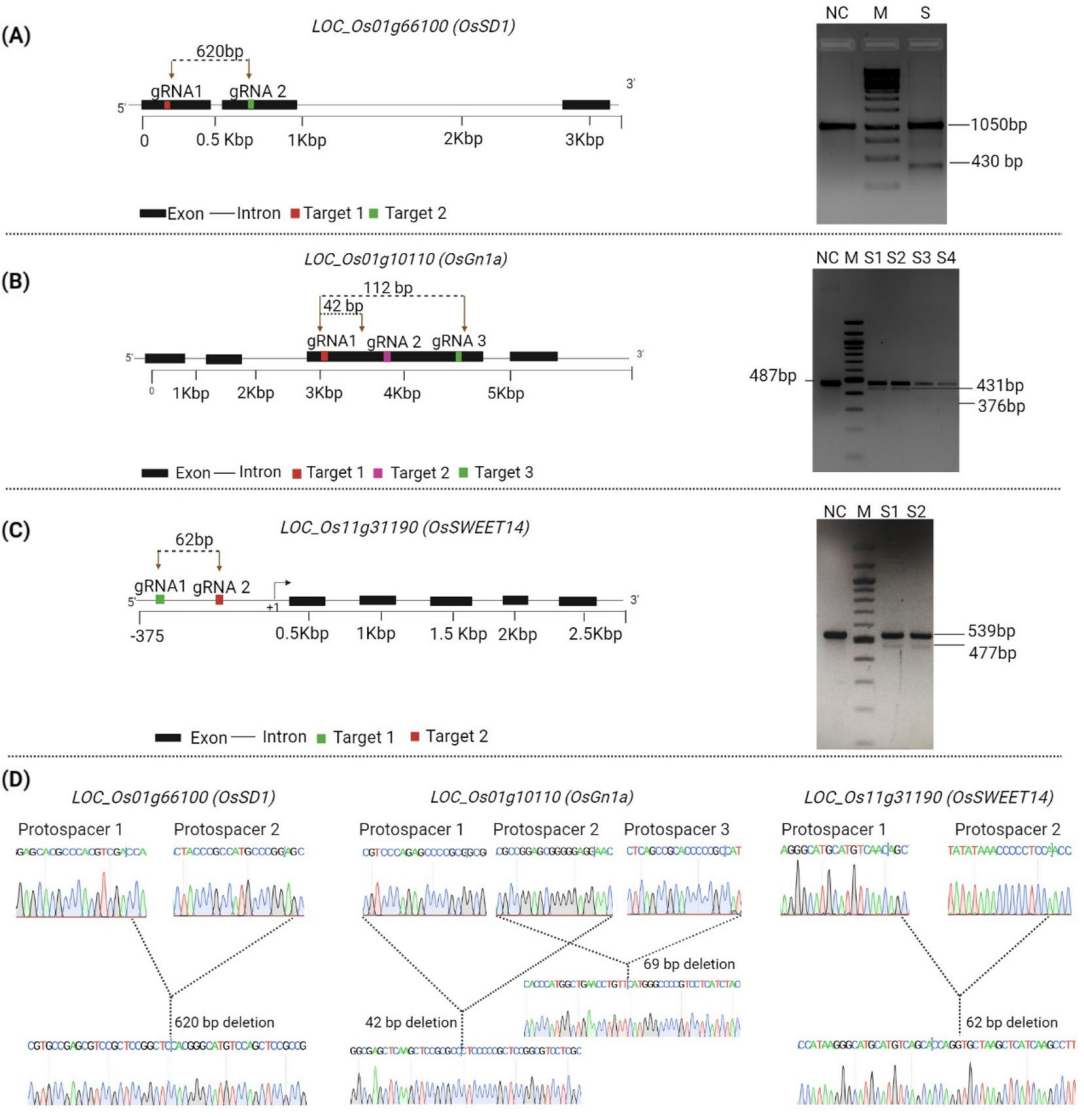
CRISPR-deletion approach in rice for rapid validation of genome editing. PCR assays and Sanger sequencing to detect CRISPR-Cas9-induced mutations in rice protoplasts targeting *OsSD1*, *OsGn1a* and *OsSWEET14* genes, respectively. **A** Images displaying the gRNA positions in the coding and non-coding regions of the *OsSD1* gene, along with the gel image depicting the PCR-amplified products from the gDNA of pRGE-SD1-GG transfected and non-transfected protoplasts. **B** Images displaying the gRNA positions in the coding and non-coding regions of the *OsGn1a* gene, along with the gel image depicting the PCR-amplified products from the gDNA of pRGE-Gn1a-GG transfected and non-transfected protoplasts. **C** Images displaying the gRNA positions in the promoter region of *OsSWEET14* gene, along with the gel image depicting the PCR-amplified products from the gDNA of pRGE-SWEET14-GG transfected and non-transfected protoplasts. **D** Images illustrating the genome editing outcomes for *OsSD1*, *OsGn1a* gene, and *OsSWEET14* promoter, respectively. Chromatograms demonstrating the protospacer and deletion position of the respective target genes. ‘S’ represents the sample number. ‘NC’ represents untransformed negative control. ‘M’ represents DNA ladder

We next validated three CRISPR vectors, namely pRGE-SD1-GG, pRGE-Gn1-GG, and pRGE-SWEET-GG for rice genome editing, using the above-described isolation and transfection protocol. The activity of these CRISPR vectors was assessed in rice protoplasts three days post-transfection. For each target gene, we used two to three sgRNAs for implementing CRISPR-deletion approach, which allows us to get indicative genome editing results from agarose gel electrophoresis. Two guide RNAs induce two double-strand breaks (DSBs), often resulting in the deletion of the intervening genomic fragment. We amplified each target locus from the genomic DNA isolated from the transfected protoplasts. Agarose gel electrophoresis revealed more than one fragment in each case, indicating successful deletion (Fig. 7A, B, and C). The lower band size was in accordance with the expected size after the deletion of the intervening region between the two DSBs. To further confirm if the lower band is due to Cas9-induced deletion or non-specific amplification, we have cloned the lower band for each target into the pGEMT-Easy vector. Sanger sequencing revealed a 620 bp deletion for *SD1* locus and 62 bp deletion for *OsSWEET14* promoter (Fig. 7D). Targeting the *OsGn1a* gene with three guides resulted in deletions of 42 bp (region between gRNA1 and gRNA2) and 112 bp (between gRNA1 and gRNA3), respectively (Fig. 7D). In all cases, a control set was included, which did not show any additional bands after amplification. Therefore, our protoplast transfection combined with the CRISPR-deletion approach can rapidly indicate successful editing only after agarose gel electrophoresis.

### Replicability of the protoplast isolation, transfection, and CRISPR-deletion protocol in dicots: *Arabidopsis* and chickpea

We then tested whether our protocol could be replicated in dicot plants like *Arabidopsis* and chickpea.

Three-week-old leaves of *Arabidopsis* (15 leaves) and chickpea (30 leaves) were used for protoplast isolation, following the same protocol standardized for rice. In *Arabidopsis*, eCaMV-GFP transfection efficiency was tested with and without the sucrose gradient step. The results showed a transfection efficiency of 62% with sucrose versus 40% without sucrose (Supplementary Fig. S5). Similarly, chickpea protoplast showed enhanced efficiency with the sucrose step compared to without sucrose (Supplementary Fig. S6).

**Fig. 8:**
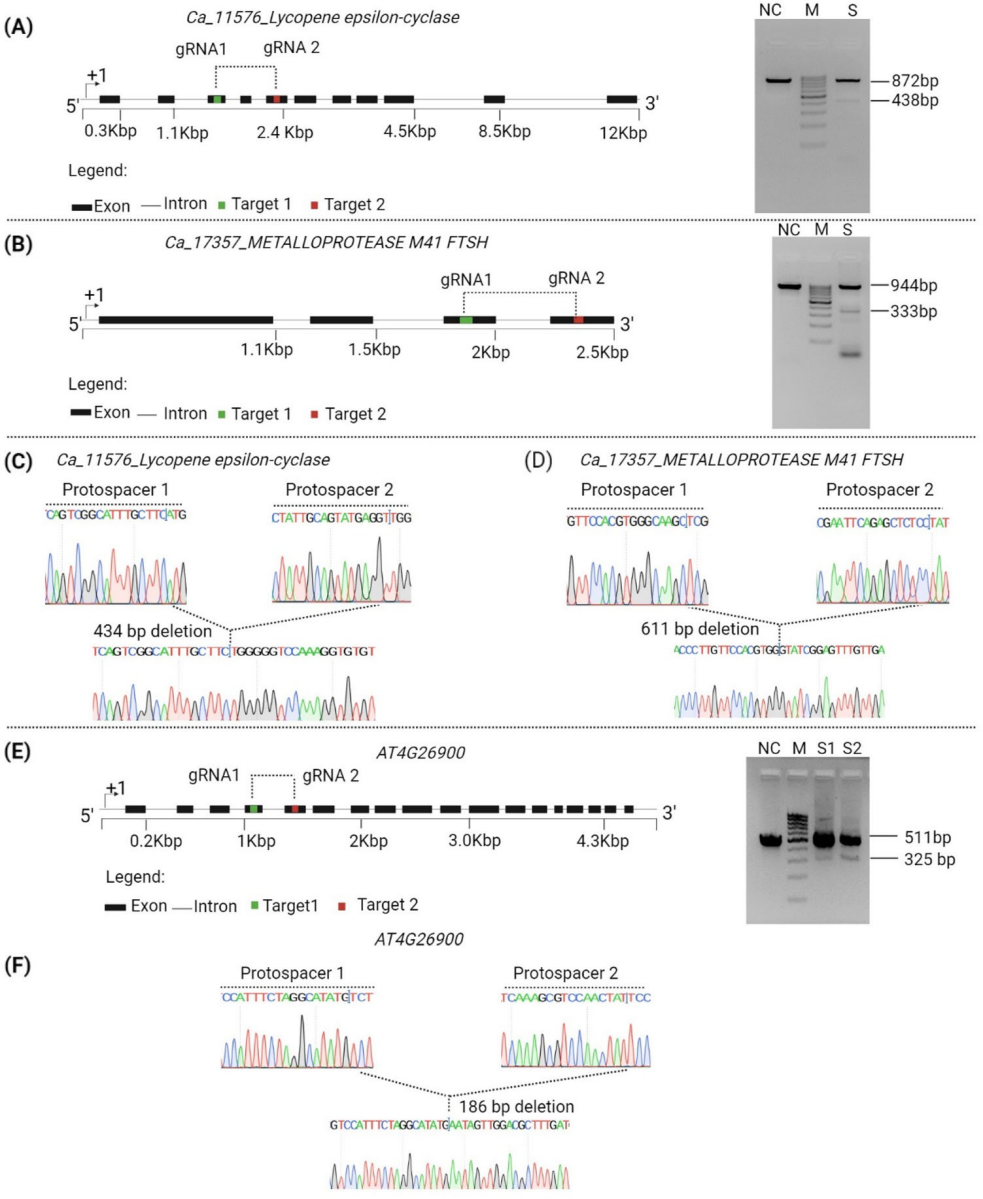
CRISPR-del approach in dicot. PCR assays and Sanger sequencing to detect CRISPR-Cas9 induced mutations in Chickpea and *Arabidopsis* protoplasts targeting the *CaLCY, CaM41, and AtGAT* genes, respectively. **A** Images displaying the gRNA positions in the coding and non-coding regions of the *CaLCY* gene, along with the gel image depicting the PCR-amplified products from the gDNA of CaLCY-GG transfected and non-transfected protoplasts. **B** Images displaying the gRNA positions in the coding and non-coding regions of the *CaM41* gene, along with the gel image depicting the PCR-amplified products from the gDNA of CaM41-GG transfected and non-transfected protoplasts. **C** and **D** Images illustrating the genome editing outcomes for *CaLCY, and CaM41* genes, respectively. Chromatograms demonstrating the protospacer and deletion position of the respective target genes. **E** Images displaying the gRNA positions in the coding and non-coding regions of the *AtGAT* gene, along with the gel image depicting the PCR-amplified products from the gDNA of AtGAT-GG transfected and non-transfected protoplast. **F** Images illustrating the genome editing outcomes for *AtGAT* gene. Chromatograms demonstrating the protospacer and deletion position of the respective target genes. ‘S’ represents the sample number. ‘NC’ represents untransformed negative control. ‘M’ represents DNA ladder

The suitability of the isolated protoplasts for genome editing was assessed with the CRISPR-deletion approach in *Arabidopsis* and chickpea. We transfected pRGEB31-GAT in *Arabidopsis* and pRGEB31-LCY, and pRGEB31-M41 in chickpea. For each target, two gRNAs were employed. Genomic DNA, isolated from the protoplasts 72 h post-transfection, was used to amplify target loci with specific primers. The amplified products showed more than one band when run on agarose gel, whereas the untransfected control showed a single band (Fig. 8 A, B, and E). Sanger sequencing of the smaller bands revealed the deletion of 434 bp in *CaLCY*, 611 bp in *CaM41*, and 186 bp in *AtGAT* genes. (Fig. 8C, D, and F). These results suggest that the protocol is, indeed, applicable to dicot plants with variable efficiency and could facilitate easy detection of genome editing when the CRISPR-deletion approach is used.

## Discussion

In the era of genome editing, the protoplast method has started playing an important role in facilitating efficient delivery of genetic material, rapid assessment of editing strategies and design, generating transgene-free mutants, and high throughput screening.

Protoplast isolation and transient transfection systems have been established in several crops, facilitating functional validation of target genes (Yoo et al. 2007; Li et al. 2016; Lin et al. 2020). Previous protocols for protoplast isolation from green tissue and calli have been established (Yoo et al. 2007; Zhang et al. 2011; Poddar et al. 2020). Although isolated protoplasts from callus are suitable for experimental studies, the procedure is time-consuming, and callus culture is susceptible to contamination. Here, we have standardized a rapid, and highly efficient protoplast isolation procedure from etiolated rice seedlings. This protocol is adaptable to other plants, as evidenced from our demonstration in dicot species, *Arabidopsis,* and chickpea. There is a direct relationship between protoplast viability and transfection efficiency. Hence, an isolation protocol that yields a high percentage of viable protoplast is always desirable for downstream analysis. Adding a centrifugation step with 0.55 M sucrose greatly facilitated accumulation of viable protoplasts, in the interface, while removing cellular debris (Figs. 1 and 2). Here, DNA may transform nonviable protoplasts, but this would not render transgene expression. As a result, transformation efficiency is reduced. The use of a sucrose step drastically reduces the percentage of dead and broken protoplasts in the isolated sample (Figs. 1 and 2). A sucrose overlaying step has been used successfully in several earlier studies for isolating protoplasts from different plants (Brandt et al. 2020; Poddar et al. 2020; Jeong et al. 2021). The sucrose step also showed significant improvement in transfection efficiency in rice protoplasts over the protocols where the step is not included (Fig. 5).

Performing protoplast isolation and transfection on the same day can be a time-consuming and demanding process. It also poses a limitation on the number of transfections that could be handled. Also, it is not uncommon that midi-prep of some plasmids may fail in the first attempt. Preserving protoplast viability for a minimum of 24 h would offer valuable flexibility to researchers, allowing them to take a break between the isolation and transfection steps. Our result showed that the isolated protoplasts can be stored in MMG solution for 24 h at RT (25 °C) with minimal loss of viability and they can be very well transfected even after 48 h (Fig. 6). An earlier study showed preserving viability of protoplast isolated from rice calli tissue (Poddar et al. 2020). Our study for the first time showed the storability of isolated protoplasts from rice seedlings. Culturing rice embryos to generate calli tissue involves the risks of contamination and is a time-consuming process. In contrast, utilizing rice seedlings as the starting material for protoplast experiments offers numerous advantages. Our protocol with a pause step between protoplast isolation from seedling and transfection would allow handling a larger number of transfections on the following day or conducting another round of midi-prep for failed constructs.

Protoplast-based transient expression systems have been employed for functional genomics studies (Lorenzo et al. 2019), and a transfection efficiency of at least 50% is considered reliable (Yoo et al. 2007). We have optimized various factors for PEG-mediated protoplast transfection. In this study, we achieved highly efficient transfection (81%) in rice protoplast isolated from 10-day-grown etiolated rice seedlings using 30 μg of plasmid DNA, 200 µL of protoplasts [stock concentration: 2 × 10^6^ cells/mL], and 20 min of incubation in a PEG solution. Notably, we have also tried with etiolated seedlings of both *Arabidopsis* and Chickpea but etiolation causes problems in plants viz. abnormal growth of both stems and leaves, weakens the cell walls, elongates the internodes with fewer leaves than normal condition. Therefore, it was difficult to obtain sufficient leaves when grown in the dark. Here, we have chosen light-grown 20–25 days old green leaves for protoplast isolation for both dicot plant species. In an earlier study, transfection efficiency was observed to be nearly 53–75% in rice (Zhang et al. 2011). Similarly, up to 73.5% efficiency was achieved in protoplast isolated from rice calli (Poddar et al. 2020). We have observed superior transfection efficiency with smaller size of plasmids (Fig. 3D–H). Similar to the present investigation, maximum transfection efficiency (75%) was recorded with small-sized plasmids, whereas larger binary plasmid obtained 45–66% efficiency (Zhang et al. 2011). Amount of plasmid DNA has a great impact on transfection efficiencies. In our study, we tested transfection efficiencies with different concentrations of plasmid DNA and observed the highest transfection frequency (81%) with 30 µg of plasmid DNA in 200 µL of protoplasts (conc. 2 × 10^6^/mL). A comparatively lower (30–60% in rice) percentage of transfected protoplasts was recorded with 10–25 µg of the plasmid DNA. In our study, we also observed that transfection efficiencies for etiolated rice protoplasts were 10–20% more compared to the green protoplasts.

Genome editing using CRISPR/Cas9 generates mutations at specific target sites. However, not all the CRISPR vectors and gRNAs are active and efficient. Given that plant regeneration using the tissue culture method in monocot such as rice and dicots is a time-consuming process, so, it becomes crucial to validate the efficiency of sgRNA before performing a stable transformation.

Our CRISPR-deletion strategy using two sgRNAs for targeting each gene only reflects the efficiency of targeted deletion and that can be easily visualized on the agarose gel. This method allows for rapid validation of sgRNAs and Cas proteins, and different editing strategies. CRISPR-induced successful mutations were deciphered using PCR, gel electrophoresis, and Sanger sequencing from transfected protoplasts after 72 h of transfection. We have demonstrated successful genome editing with our protocol in all three target genes by detecting additional smaller PCR bands with the WT band (Fig. 7A–C). The appearance of a smaller band is an indication that both the sgRNAs are capable of inducing DSB causing deletion of the intervening region. The result was confirmed by Sanger sequencing. However, it should be noted that in this context, the visualization of a smaller band on the agarose gel only reflects deletion efficiency. If other types of small indels occur independently at one or both of the guides, the band with mutations cannot be distinguished from the wild-type band in agarose gel.

Genome editing using CRISPR/Cas9 has been demonstrated successfully in several legumes (Wang et al. 2016; Meng et al. 2017; Ji et al. 2019; Liu et al. 2019). In spite of this accomplishment, the recalcitrant nature of in vitro genetic transformation and stable regeneration of chickpea remain a serious bottleneck for the implementation of genome editing tools in this important and nutrient-rich crop (Das Bhowmik et al. 2019). Protoplast transformation using CRISPR-Cas9 targeting drought-associated genes was reported recently (Badhan et al. 2021). Here, we have demonstrated the applicability of our isolation and transfection protocol in chickpeas and Arabidopsis. Similar to rice, the CRISPR-deletion approach yielded expected deletion in one *Arabidopsis* gene and two chickpea genes (Fig. 8C, D, and F). A handful of reports are available that show editing experiments in chickpeas (Gupta et al. 2023). This protocol will facilitate researchers to undertake genome editing in chickpea. Thus, our work offers an effective strategy to easily and rapidly validate the CRISPR reagent sites, endorsing their application for genome editing in both monocots and dicots.

Standardisation of protoplast regeneration will help avoid potential problems, such as chimerism which can be seen during transformation using *Agrobacterium* or particle bombardment (Reed and Bargmann 2021). Regenerated plants from protoplast are of single-cell origin and avoid such problems. Additionally, protoplast transformation efficiencies are higher compared to other transformation processes, increasing the likelihood of successful editing events in plants transformed with editing reagents. Our protocol should be compatible with other CRISPR systems, such as CRISPR-Cas12a and precise base editors and prime editors (Zhang et al. 2019; Molla et al. 2021). This method could also be used for RNP transfection for generating transgene-free mutants (Zhang et al. 2021). Encouragingly, recent studies showed that RNP delivery of CRISPR-Cas12a could generate nearly 100% genome editing efficiency in protoplasts from different plant species (Zhang et al. 2022), which translated to high-efficiency genome editing in regenerated plants such as citrus (Su et al. 2023). We hope that the protocol described here will encourage others to adopt it for application in their favourite plant systems which will open many unknown potentials.

## Conclusions

This present investigation developed an improved process of protoplast isolation from etiolated rice seedlings and green leaves from *Arabidopsis* and chickpea. Using sucrose gradient after enzymatic digestion, isolated protoplasts are larger in size, with an increased surface area to volume ratio, which improves the transfection efficiency. It is noteworthy that our protocol allows a pause point between isolation and transfection, offering high flexibility. In addition, we demonstrate that protoplasts isolated using this protocol are suitable for transfection and transient expression analysis and, most importantly effective for in vivo validation of gRNAs used for CRISPR-Cas based genome editing for both monocot and dicot plants.

## Materials and methods

### Plant material and growth condition

#### Rice

Rice cultivars used for protoplast isolation were indica (*Oryza sativa* L. indica v Nua kalajeera) and Japonica (*Oryza sativa* L. subsp. japonica cultivar Kitaake). At least 80 dehusked rice seeds were surface sterilized using 30 mL of 70% (v/v) ethanol in a 50 mL conical flask with vigorous shaking for 30 to 40 s, and then washing with 30 mL of sterile water, followed by further washing in 30 mL of commercial bleach (4% hypochlorite) added with 0.1% Tween-20 for 25 min on a magnetic stirrer. The bleach solution was discarded and the seeds were rinsed five times with sterilized water. After the final rinse, seeds were poured onto the sterilized filter paper, dried, and subsequently transferred onto a sterile 75 mL full MS medium in a 400 mL bottle (Axiva, Cat. No. TCB 400). The bottles were wrapped with aluminum foil and incubated in the dark at 28 °C for 10–12 days.

#### Arabidopsis thaliana

Seeds of *Arabidopsis thaliana* (*A. thaliana*) Col-0 (20 mg) were placed into a 1.5 mL microcentrifuge tube, rinsed with 70% ethanol for 1 min through pipetting, and centrifuged at 300 g for 20 s. After centrifugation, the supernatant was discarded through pipetting. A commercial bleach solution containing 0.1% Tween-20 was added to the tube. Another low-speed centrifugation was performed, followed by washing the seeds with sterile water, at least 3–4 times. The seeds were dried and then placed on half-strength MS media. The seed-containing plate was incubated at 4 °C for two days in the dark, after which the plate was placed under light., and seeds were germinated at 22 °C, with a 16-h light and 8-h dark cycle, in a growth chamber. After one week, plants were subcultured on full-strength MS media and, finally, after 3 weeks, the leaves were used for protoplast isolation.

#### Chickpea (*Cicer arietinum L.*)

Twenty seeds of chickpea (*Cicer arietinum* L. GNG2144) were placed in a 100 mL conical flask and thoroughly rinsed with autoclaved distilled water. Then 50 mL of 70% ethanol was added on seeds for 40 s and rinsed with autoclaved distilled water. Subsequently, 30 mL of sodium hypochlorite solution containing 4–5 drops of 1% Tween-20 was added. The flask was then placed on a magnetic stirrer for 20 min. Afterward, the bleach solution was gently discarded, and the seeds were rinsed multiple times with autoclaved distilled water until all traces of the bleach solution were removed. The surface-sterilized seeds were soaked in distilled water overnight, and the following day, they were rinsed twice with autoclaved distilled water. These seeds were then transferred onto sterilized filter paper, dried completely, and subsequently placed into a sterile 75 mL full MS medium within a 400 mL bottle (Axiva, Cat. No. TCB 400). These bottles were maintained under a 16-h light and 8-h dark cycle at 25 °C for a period of 3 weeks.

### Vector construction

#### Rice

The CGBE1 plasmid vector (Addgene Plasmid # 140252) harboring the eGFP sequence was used as a template for PCR. The primer set, 242F1 & 243R1 harboring BstBI and XbaI sites, respectively, was used to amplify the GFP coding sequence. Purified PCR products were cloned into a pGEMT-Easy vector for sequence confirmation.

For modifying the GFP coding sequence to BFP, changing only two amino acids is required at the 65th and 66th positions. To convert the GFP sequence to the BFP sequence, site-directed mutagenesis in the GFP coding sequence was performed through overlap extension PCR using 242F1-245R2 and 244F2-243R1 primer pairs. pGEMT-Easy-GFP plasmid was used as a template for amplifying fragment 1 (212 bp) with 242F1-245R2 primer pairs and fragment 2 (544 bp) with primer set 244 F2-243R1 using Q5 polymerase (NEB, USA). Primers, 244F2 and 245R2 harboured the intended modification for site-directed mutagenesis. Overlap extension PCR was performed with two fragments to amplify the 738 bp BFP sequence. The product was purified, and cloned in pGEMT-Easy, and sequence confirmed. Finally, GFP and BFP were cloned into pRGE32 (Xie et al. 2015) and pRGEB32 (Xie et al. 2015) vector backgrounds downstream of *OsUbi* promoter for transient expression analysis in protoplasts.

Promoter sequence of rice *SWEET14*, coding sequence of *GA20 oxidase 2* (*OsSD1*) and *OsGn1a* genes were cloned and sequenced from both Indica and Japonica rice varieties used in the study. For editing, two or three guides for each of the target genes were designed. Polycistronic tRNA-gRNA (PTG) system was used to clone two or more guides in tandem (Xie et al. 2015). *SWEET14*-PTG contains two guides targeting TalC and AvrXa7/PthXo3 effector binding sites within the *OsSWEET14* promoter. We assembled two guides for the *OsSD1* coding sequence and three guides for *OsGn1a* coding site. For constructing PTG, individual fragments were amplified by PCR using Q5 polymerase (NEB, USA) with specific primer sets taking pGTR (Addgene Plasmid # 63143) vector as the template, and then assembled through Golden Gate. The assembled PTGs were cloned into a pGEMT-Easy vector and the sequences were confirmed through Sanger sequencing. After sequence confirmation, the fragments were ligated into the BsaI site of pRGE32 to generate pRGE32-SWEET14-GG, pRGE32-SD1-GG, and pRGE32-Gn1a-GG.

#### Arabidopsis thaliana

The vectors eCaMV-GFP and *AtUbi10*-GFP were used for *Arabidopsis* GFP transfection. The vectors were prepared by modifying the pRGE32-GFP vector. The eCaMV35S promoter was amplified from pH-Mono-MAD7 (Addgene, Plasmid # 170134) using 621F and 622R primer pairs having BstBI and PstI restriction sites, respectively. Similarly, the *AtUbi10* promoter was amplified from the genomic DNA of *Arabidopsis thaliana* using primer sets 623F and 748R harboring BstBI and PstI restriction sites, respectively. Both the promoters were sequenced and used to replace *OsUbi* in the pRGE32-GFP vector to construct *eCaMV35S::GFP*, and *AtUbi::GFP*. These two constructs were used for GFP transfection in *Arabidopsis*. For editing, we replaced the CaMV*35S* promoter with the eCaMV*35S* promoter in pRGEB31 (Xie and Yang 2013). The new construct was termed pRGEB31-eCaMV*35S*. Amplification of *AtU6-26* promoter from genomic DNA of *Arabidopsis* was performed using 618F and 846R primer sets, and the product was sequenced. The *OsU3* promoter of pRGEB31-eCaMV*35S* was replaced by *AtU6-26.* Two guides were designed to target the *glutamine amidotransferase* (*AtGAT*) gene and PTG was assembled using Golden Gate assembly as described in the case of rice. The assembled fragments were Sanger sequenced and finally subcloned in the BsaI restriction site of the pRGEB31-eCaMV35S-AtU6 vector. The resulting construct was named pRGEB31-GAT.

#### Chickpea (*Cicer arietinum L.*)

Fragments of *Lycopene epsilon cyclase* (*CaLCY*) and *METALLOPROTEASE M41 FTSH* (*CaM41*) were amplified using 430-Ca_LCY-F, 431-Ca_LCY-R, 414-CaM41-F, and 415-CaM41-R primer sets, respectively, and verified with Sanger sequencing. Two guides were designed for each of the target genes using CHOPCHOP software (Labun et al. 2019), and the PTGs were assembled using the Golden Gate assembly. The assembled fragments were subsequently cloned into a TA cloning vector, and confirmed with Sanger sequencing. Both the assembled fragments were separately subcloned into the BsaI restriction site of the pRGEB31 vector. The resulting vectors were designated as pRGEB31-LCY and pRGEB31-M41.

### Protoplast isolation and density evaluation from rice

Protoplasts were isolated from rice tissue following a previously published protocol with several modifications (Tang et al. 2016). Protoplast isolation from rice seedlings (approximately 10–12 days old) was initiated by cutting the stem of etiolated rice seedlings (60 seedlings per 10 mL enzyme solution) perpendicular to the direction of growth. Leaf blades and roots including the base of the prophyll were removed by sterile scissors from the etiolated seedlings. Stems of the seedlings were chopped with single-edge razor blades (Hyde Tools 13,125 Single Edge Razor Blades) with approximately 0.5–1 mm in diameter perpendicular to the growth of the seedlings. These strips were then transferred to a sterile 100 mL conical flask containing 10 mL of 0.6 M mannitol (Supplementary Table S1) and incubated in darkness for 10 min. The mannitol was carefully removed using a 10 mL sterile pipette fitted with a sucker and 10 mL of protoplast isolation buffer (Supplementary Table S2) was added to the cut strips. Afterward, cut strips were vacuum infiltrated in protoplast isolation buffer for 5 h at 25 °C in the dark with gentle shaking on an orbital shaker at 40 rpm (see supplementary note 1) for 5 h. After 5 h of incubation, the protoplast solution was shaken at 80 rpm for 10 min followed by gentle hand shaking for an additional 2 min alternating between clockwise and anticlockwise rotation.

Protoplasts were filtered using a 100 µm Cell Strainer, (Corning^®^, Cat.No. CLS431752-50EA) pre-wetted with 1 mL of W5 buffer (Supplementary Table S3). To collect the remaining cut strips, an additional 10–20 mL of W5 buffer was added into the conical flask, swirled gently by the hand, and poured onto the strainer. A gentle press was applied to the inner surface of the strainer to facilitate the release of more protoplasts. A second round of filtration was performed with a 40 µm Cell Strainer (Corning^®^, Cat.No. CLS431750-50EA) in a fresh 50 mL falcon tube (see supplementary note 2). The centrifugation was performed at 100 *g* for 7 min in a swing bucket rotor (acceleration and deceleration were set at 6 or 7), and the supernatant was carefully removed. The visible pellet was resuspended in 5–7 mL of W5 buffer by gentle tapping.

The homogeneous solution was transferred to a sterile 14 mL round bottom tube (Nunc^™^ 14 mL Round-Bottom Tube, Cat.No. 150268) (see supplementary note 3), and centrifuged at 100 *g* for 7 min. The supernatant was pipetted off carefully, and the pellet was resuspended in 6 mL of a 0.55 M sucrose solution (Supplementary Table S6). At that point, 2 mL of W5 buffer was carefully added on top of the sucrose-protoplast solution to minimize the mixing of these two solutions (the interface should be clearly visible), and centrifuged for 30 min at 100 g. After centrifugation, a band of protoplasts was clearly visible at the interface (Fig. 4 and supplementary Fig. 1), and the tube was incubated on a benchtop for 15–30 min to facilitate the accumulation of more protoplasts. Using a 200 µL pipette with cut tips (see supplementary note 4), a band of protoplasts was transferred from the interface to a new round-bottom tube (typically ~ 1 mL of protoplasts) and checked under the microscope using a Hemocytometer. The visible concentrated round protoplasts at this step were further washed by adding 5 mL of W5 buffer using centrifugation at 100 g for 5 min. After that, the supernatant was carefully removed leaving 0.25 mL, and further counted using a hemocytometer. Finally, MMG buffer (Supplementary Table S4) was added to obtain a titer of 2 × 10^6^ protoplasts per mL. A detailed protocol is given in supplementary file.

### Protoplast isolation and density evaluation from *Arabidopsis* and *Chickpea*

Protoplast isolation from *Arabidopsis* and chickpea leaves was performed as detailed below. Three-week-old leaves of *Arabidopsis* (15 leaves) and chickpea (30 leaves) were chopped with single-edge razor blades into 0.5–1 mm strips. The strips were transferred in a sterile 100 mL conical flask containing 10 mL of 0.6 M mannitol and kept in the dark for 10 min. Mannitol was removed, and 10 mL protoplast isolation buffer was added to the conical flask. The conical flask was incubated for 5 h at 25 °C in the dark with gentle shaking on an orbital shaker at 40 rpm with vacuum infiltration. The following steps are similar to those described for rice. Composition of different solutions, buffers and reagent set up are given in the supplementary file.

### Protoplast viability check

The viability of protoplasts from rice, *Arabidopsis*, and chickpea was evaluated separately by using two different dyes. Firstly, 1% (w/v) Evans Blue staining was performed following a previously described protocol (Gaff and Okong’o-ogola 1971). Secondly, 0.01% of fluorescein diacetate (FDA) was used to stain protoplasts (Williams and Co 1972). Briefly, 5 µL of 1% Evans Blue was added to 20 µL of the protoplast solution. Evans Blue-stained protoplasts were visualized under a bright field using a Leica DMi8 fluorescence microscope with N.A. 1.25 (Leica Microsystems Inc., Buffalo Grove, IL). LAS X software was utilized for image acquisition and analysis. The protoplast viability was also assessed by adding 1 µL of 0.01% of FDA dissolved in acetone to 20 µL of protoplasts. The samples were visualized using a Leica DMi8 fluorescence microscope with a 480–510 nm excitation filter and a 535–585 nm emission filter. Data were recorded for five consecutive fields along with their corresponding bright field images (Grey). The green fluorescence was observed using a FITC filter excited at 488 nm with emission at 505–525 nm. The percentage of viable and non-viable protoplasts was calculated by dividing the number of unstained and stained cells by the total number of cells visible in each microscopic field.

### PEG-mediated protoplasts transfection for rice, *Arabidopsis* and chickpea

The isolated protoplast solution was diluted with MMG buffer to obtain a working stock of 2 × 10^6^/mL for transfection. 200 µL rice protoplast solution was transferred into 2 mL round-bottom microcentrifuge tubes. 30 µL of plasmids at a concentration of 1000 ng/µL were added and mixed gently by tapping. Different amounts of total DNA were used to standardize the optimum concentration and different sets were prepared for pRGE32-GFP, pRGE32-BFP, and pRGEB32-GFP reporter plasmids, and pRGE32-SWEET14-GG, pRGE32-SD1-GG, and pRGE32-Gn1-GG plasmids with three biological replicates. Plasmids were prepared using a Qiagen Plasmid plus midi kit (Qiagen, Cat. No. 12943). The protoplast and plasmid mixtures were incubated at room temperature for 10 min. Subsequently, 230 µL of freshly prepared PEG-calcium chloride transfection buffer (see solution composition, Supplementary Table S5) was added to each tube wall in a dropwise manner, gently mixed by inversion, and incubated for 20 min at room temperature.

Meanwhile, a 12-well culture plate (NEST^®^, Cat. No. 712001) was made ready by adding 80 µL of 5% calf serum (Gibco, Cat. No. 16170–086) in each well and spreading it using a 100 µL pipette throughout the inner wall of each well. Then, 1 mL of W5 buffer was added to each well. After the 20-min incubation, 900 µL of W5 buffer was added to each 2 mL round bottom tube, and the solution was gently mixed by inverting four times. The tubes were centrifuged at 100 *g* for 5 min to pellet the protoplasts using a swing bucket rotor. Then, half of the supernatant (680 µL) was carefully removed using a 1 mL pipette, and the remaining solution was mixed by gentle inversion for 4 times. The protoplast mixture was transferred to the 12 well culture plates. The plates were sealed with parafilm, wrapped in aluminum foil, and incubated at 32 °C in the dark for 72 h with gentle shaking at 25 rpm. The transfection procedure for *Arabidopsis* and chickpea was similar to that used for rice with two modifications: (1) the use of a higher plasmid concentration, 45 µg, and (2) a shorter 15-min incubation after mixing the protoplasts with the plasmid DNA. The protoplasts isolated from *Arabidopsis* were transfected with pRGEB31-GAT, eCaMV-GFP, and AtUbi10-GFP plasmids. Similarly, chickpea protoplasts were transfected with the constructs viz. pRGEB31-LCY, pRGEB31-M41, and eCaMV-GFP.

### GFP and BFP detection using fluorescent microscopy

Green and blue fluorescent signals were seen after 16–18 h post-transfection and gained peak at 48 h. GFP and BFP fluorescence was visualized using a Leica DMi8 fluorescence microscope (Leica Microsystems Inc., Buffalo Grove, IL) from five consecutive fields as well as their corresponding bright field images (Grey) from each replicate. GFP fluorescence was excited at a 488 nm filter and detected using a 505–525 nm emission filter. In the case of BFP, a 351–363 nm was used for excitation, and emission was recorded using a 460 nm filter.

### Storage of protoplasts for including a breakpoint

Rice protoplasts obtained from the sucrose gradient were preserved using MMG solution in a sealed 14 mL centrifuge tube (Thermo Scientific, Cat. No. 150268). The tube was covered with Parafilm and enveloped in aluminium foil for storage at room temperature (RT, 25 °C) and on ice. At intervals of 24 h (1, 24, 48 h), protoplasts underwent viability testing and transfection with a GFP-containing vector using PEG-CaCl_2_-mediated transfection, as previously described. Viability was assessed through FDA staining, and transfection efficiency was determined by counting GFP-positive protoplasts.

### Validation of CRISPR-Cas9 vectors in rice, *Arabidopsis,* and chickpea protoplasts with a CRISPR deletion approach

Genomic DNA was extracted from the transfected protoplasts after three days. Target regions were amplified using high-fidelity Q5 DNA polymerase (New England Biolabs, USA). CRISPR deletions were visualized via agarose gel electrophoresis of PCR products. PCR amplicons were purified, cloned into a TA vector and then transformed in *E. coli.* The transformants were screened by colony PCR using M13F and M13R and also with gene-specific primer sets described earlier. The positive clones were subjected to CAPS assay and Sanger sequenced for verification.

### Supplementary Information

Below is the link to the electronic supplementary material.

Supplementary file1 (DOCX 5660 KB)

## Abbreviations

CRISPR-Cas9: Clustered regularly interspaced short palindromic repeats (CRISPR)- CRISPR-associated protein 9 (Cas9)
PEG: Polyethylene glycol
GFP: Green fluorescent protein
BFP: Blue fluorescent protein
FDA: Fluorescein diacetate
PTG: Polycistronic-tRNA-gRNA
Indels: Insertions and deletions
DSBs: Double-strand breaks
gRNA: Guide RNA
sgRNA: Single guide RNA
*OsSD1*: Rice *Semi Dwarf 1*
*OsGn1a*: Rice *Grain number 1a*
*AtGAT*: Arabidopsis *glutamine amidotransferase*
*CaLCY*: *Chickpea Lycopene epsilon cyclase*
*CaM41*: *Chickpea METALLOPROTEASE M41 FTSH*
