# Supplementary material for: Optimized protoplast isolation and transfection with a breakpoint: accelerating Cas9/sgRNA cleavage efficiency validation in monocot and dicot
Source: aBIOTECH. 2024 Apr 15;5(2):151–68. doi: 10.1007/s42994-024-00139-7 (PMC11224192; doi:10.1007/s42994-024-00139-7)
Supplement: Supplementary file 1 — Supplementary file1 (DOCX 5660 KB) [file 42994_2024_139_MOESM1_ESM.docx]

**Optimized protoplast isolation and transfection with a breakpoint: Accelerating Cas9/sgRNA cleavage efficiency validation in monocot and dicot**

Debasmita Panda ^1,2^, Subhasis Karmakar ^1^, Manaswini Dash ^1^, Swagat Kumar Tripathy^1^, Priya Das^1^, Sagar Banerjee^1^, Yiping Qi^3^, Sanghamitra Samantaray^1^, Pradipta Kumar Mohapatra^2^, Mirza J Baig^1*^, Kutubuddin A. Molla ^1*^

^1^ ICAR National Rice Research Institute, Cuttack, Odisha 753006, India

^2^ Department of Botany, Ravenshaw University, Cuttack, Odisha 753003, India

^3^ Department of Plant Science and Landscape Architecture, University of Maryland, College Park, MD 20742, USA

*Corresponding author

**Supplementary data and tables**

**Content**

- **Stepwise protocol of protoplast isolation and transfection…………………2**
- **Composition of different solutions, buffers and reagent set up……………7**
- **Supplementary notes………………………………………………………...11**
- **Table S7: Primers used for this study…………………………………….. 12**
- **Supplementary figures S1-S6………………………………………………14**
- **Troubleshooting……………………………………………………………. 20**

**Supplementary data and tables**

**Stepwise protocol of protoplast isolation and transfection**

**Isolation of protoplast**

Protoplast isolation starts with the enzymatic digestion of rice seedlings to break down the cell walls, releasing the naked protoplasts from intact tissues. The protoplast solution is then filtered through a cell strainer to remove the undigested and large tissue debris, washing to remove the smaller pieces, sucrose gradient to select live and viable large protoplast, and finally dilute to make suitable concentration for downstream applications. The detailed stepwise protocol for rice protoplast isolation is presented below. The entire procedure is performed under aseptic conditions. We also do not turn on the light in the laminar airflow or workbench during the isolation procedure.

**Timing**: The growth of dark-grown albino rice seedlings requires a 10 to 12-day period. The isolation of complete protoplasts, from digestion to transfection-ready protoplasts takes ~8 hours.

1. Surface sterilize around 80 dehusked rice seeds in a 50 mL conical flask with 30 mL of 70% ethanol for 40 seconds. Wash the seeds in sterile water. Add 30 mL of 4% hypochlorite solution with 0.1% Tween-20 to the conical flask and stir in magnetic stirrer for 25 minutes. Then, rinse the seeds five times with autoclaved distilled H_2_O to remove any trace of hypochlorite. Air dry the seeds on a sterile filter paper. Subsequently, transfer them to a 400 mL bottle (Axiva, Cat. No. TCB 400) containing 75 mL MS medium. Incubate in the dark at 28 °C for 10-12 days to grow etiolated seedlings.
2. Remove leaf blades and roots, including the base of the prophyll, from etiolated seedlings with sterile scissors. Chop stems of the seedlings with single-edge razor blades (Hyde Tools 13125). Make the chopped pieces 0.5-1 mm in length.

**Note**: We use the lid of a sterile disposable Petri plate as a chopping base.

1. Transfer the cut strips to a sterile wide-mouth 100 mL conical flask containing 10 mL of 0.6 M mannitol (Supplementary Table S1) and incubate in the dark for 10 minutes. Wrapping the conical flask with aluminium foils would suffice.
2. Carefully remove the mannitol using a 1 mL pipette. Add 10 mL of protoplast isolation buffer (also known as enzyme solution) (Supplementary Table S2) to the cut strips. Vacuum infiltrate the cut strips in a protoplast isolation buffer for 5 hours at 25 °C in the dark with gentle shaking on an orbital shaker at 40 rpm.

**Note**: Ensure all cut strips are submerged. Periodically (every 30 mins) pause the vacuum and gently shake the conical to resubmerge the floating strips. For accommodating more cut strips, an additional volume of protoplast isolation buffer could be added.

1. After 5 hours of digestion, shake the conical with the protoplast solution at 80 rpm for 10 minutes. Then gently shake the conical with your hand for an additional 2 minutes, alternating between clockwise and anticlockwise rotation. Check the quality of the protoplast by taking 5-10 µL of solution in a haemocytometer. We use cut tips to draw the solution.

**Note:** To prepare cut tips, cut approximately 0.5 cm from the lower end of the tips and then expose them to a flame to blunt the ends. If incomplete digestion is observed, incubation for an additional 1 hr is required.

1. Filter the protoplast solution using a 100 µm Cell Strainer, (Corning®, Cat. No. CLS431752-50EA) to a 50 mL centrifuge tube (Supplementary table S3). A second round of filtration was performed with a 40 µm Cell Strainer (Corning®, Cat. No. CLS431750-50EA) in a fresh 50 mL falcon tube (see supplementary note 2).

**Note**: In case a cell strainer is not available, two layers of 37 µm nylon mesh can be used to filter protoplasts. 2-3 mL W5 solution should be used to prewet the nylon mess. If the pore size is bigger, use more layers of nylon mess.

Both types of cell strainer (100 µm and 40 µm) should be pre-wetted with 1 mL of W5 buffer before use.

1. Centrifuge the filtrate at 100 g for 7 minutes in a swing bucket rotor with a set acceleration and deceleration at 6 or 7. Carefully remove the supernatant with a pipette. Resuspend the pellet in 5-7 mL of W5 buffer by gentle finger tapping to prepare a homogeneous solution. Transfer the entire solution to a sterile 14 mL (Nunc™ 14mL Round-Bottom Tube, cat.no. 150268) round-bottom tube**.**

**Note**: Do not try to remove supernatant completely. A vertical swing bucket rotor is preferred over a fixed-angle rotor. Always add W5 solution through the tube wall, not directly into the pellet.

1. Centrifuge the filtrate at 100 g for 7 minutes in a swing bucket rotor with the same setting. Carefully discard the supernatant and resuspend the pellet in 5-7 mL of W5 buffer by gentle tapping until the solution becomes homogenous. Transfer the entire solution to a new 14 mL round bottom tube.
2. Centrifuge the round bottom tube with protoplasts at 100 g for 7 minutes. Carefully discard the supernatant and resuspend the pellet in 6 mL of a 0.55 M sucrose solution (Supplementary table S6) by gently tapping/swirling the tube against the floor of laminar airflow. Add 2 mL of W5 buffer slowly on top of the sucrose-protoplast solution to minimize the mixing of these two solutions (the interface should be visible). Centrifuge the tube for 30 minutes at 100 g. A band of protoplasts is visible at the interface. Incubate the tube on a benchtop for an additional 15-30 minutes to facilitate the accumulation of more protoplasts at the interface.
3. Transfer the band of protoplasts (typically ~1 mL of protoplasts) from the interface to a new 14 mL round-bottom tube with a 200 µL cut tip.

**Note:** Be cautious to prevent the healthy protoplast interface from coming into contact with sucrose.

1. Wash the isolated protoplast further by adding 5 mL of W5 buffer and centrifuge at 100 g for 5 minutes. Carefully remove the supernatant; do not try to remove it completely and leave around 0.25 mL. At this stage, count the protoplast using a hemocytometer. Finally, add the required volume of MMG buffer (Supplementary Table S4) to obtain the titter of 2 x 10^6^ protoplasts per mL.

**Note:** For counting and calculation of cell numbers, this easy to use tool may be followed. <https://www.hemocytometer.org/hemocytometer-calculator/>

We use the same protocol for Dicot (*Arabidopsis* and chickpea) mesophyll protoplast isolation using green leaves (three-week-old 15 leaves of *Arabidopsis,* and 30 leaves for chickpea).

**Protoplasts viability check**

Check the viability of protoplasts by staining with 1% (w/v) Evans Blue and/or with 0.01% of fluorescein diacetate (FDA). Then check viable and non-viable protoplasts under the bright field for Evans blue-stained protoplasts and under a fluorescence microscope for FDA-stained protoplasts. Live cells will not be stained by Evans blue, while live cells will fluorescence when stained with FDA.

**Storage:** In this stage, the isolated protoplasts can be stored in MMG solution at room temperature for up to 24-48 hours, if required. However, fresh transfection is always preferable.

**PEG-mediated protoplasts transfection for Rice**

Once the concentration of the protoplast solution is made 2 x 10^6^ protoplasts per mL, it is ready for transfection.

**Timing:** From PEG-mediated transfection to GFP visualization and checking for genome editing, it takes three days.

1. Dilute the protoplast solution with MMG buffer to obtain a working stock of 2 x 10^6^/mL for transfection.
2. Take 30 µL of transfection grade plasmid at a concentration of 1000 ng/µL in a 2mL round bottom microcentrifuge tube (BR BIOCHEM, Life sciences, Cat. No. B2MCT). Then add 200 µL of rice protoplasts solution into the plasmid-containing tube and mix gently by tapping.

**Note:** We use GFP cassette Plasmid as a positive control to check the transfection efficiency. Use a similar distinct set for your other plasmids.

1. Prepare three biological replicates with each of your plasmids. We use a Qiagen Plasmid plus midi kit (Qiagen, Cat. No. 12943) to isolate transfection-grade plasmid DNA. Other kits of your choice can also be used.
2. Incubate the protoplast and plasmid mixtures at room temperature for 10 minutes. After that, add 230 µL of freshly prepared PEG-calcium chloride transfection buffer (Supplementary table S5) to the tube wall dropwise. Gently mix the PEG-CaCl_2_ by slow inversion, and incubate the mixture for 20 minutes at room temperature.
3. Meanwhile, prepare a 12-well culture plate (NEST®, Cat. No. 712001) by adding 80 µL of 5% calf serum (Gibco, Cat. No. 16170-086) in each well and spread it using a 100 µL pipette throughout the inner wall of each well. Then add 1 mL of W5 buffer to each well.
4. Add 900 µL of W5 buffer to each 2 mL round bottom tube containing transfection mix and gently mix the solution by inverting it four times.
5. Centrifuge the tubes at 100 g for 5 minutes to pellet the protoplasts using a swing bucket rotor.
6. Carefully discard half of the supernatant (680 µL) using a 1 mL pipette, and mix the remaining solution by inverting the tube gently.
7. Transfer the protoplast mixture to the 12 well culture plates using a pipette with cut tips, from each tube to a pre-marked well.
8. Seal the culture plates with Parafilm, wrap them in aluminum foil, and incubate at 32 °C in the dark for 72 hours with gentle shaking at 25 rpm.
9. After 48 hours, check the transfection efficiency by visualizing and counting GFP-positive protoplasts. For downstream analysis (for example, genome editing efficiency evaluation), genomic DNA is isolated after 72 hours of transfection.

**Note:** Even after 16 hours of transfection, GFP-expressing cells could be visualized.

The transfection procedure for Arabidopsis and chickpea was similar to that used for rice with two modifications: (a) use a higher plasmid concentration (45 µg) and (b) incubate for 15 minutes (instead of 10 minutes used for rice) after mixing the protoplasts with the plasmid DNA.

**Composition of different solutions, buffers and reagent set up**

**Reagents setup:**

**Stocks**

- **0.8 M Mannitol:** Dissolve 14.576 g of D-mannitol (Sigma-Aldrich, Cat. No. M1902-500G) in 75 mL of autoclaved distilled H_2_O. Thoroughly mix it, and then adjust the volume up to 100 mL. Filter sterilize it using a 0.22 µm filter (Corning, Cat. No. 431229).
- **0.2 M MES buffer:** Dissolve 4.265 g of MES (2-(N-morpholine) ethanesulfonic acid) (Sigma-Aldrich, Cat. No. RES0113M-A7) in 70 mL of autoclaved distilled water. Thoroughly mix it and maintain the pH to 5.7 by adding 10N NaOH. Adjust the volume up to 100 mL, and filter sterilize it using a 0.22 µm filter.
- **1M CaCl_2_:** Dissolve14.70 g of CaCl_2_. 2H_2_O (Sigma-Aldrich, Cat. No. C3306-500G) in 70 mL of distilled water. Adjust the volume up to 100 mL and autoclave it.
- **2M KCl:** Dissolve 14.91 g of KCl (Sigma-Aldrich, Cat. No. P9541-500G) in 70 mL of distilled water. Adjust the volume up to 100 mL and autoclave it.
- **5M NaCl:** Dissolve 29.22 g of NaCl (Sigma-Aldrich, Cat. No. 71376-1Kg) in 70 mL of distilled water. Adjust the volume up to 100 mL and autoclave it.
- **1M MgCl_2_:** Dissolve 20.33 g of MgCl_2_.6H_2_O (Sigma-Aldrich, Cat. No. 63068-250G) in 70 mL of distilled water. Adjust the volume up to 100 mL and autoclave it.
- **1M D-Glucose:** Dissolve 18.01 g of D-Glucose (Sigma-Aldrich, Cat. No. G7021-1KG) in 70 mL of distilled water. Adjust the volume up to 100 mL and autoclave it.
- **1M Sucrose:** Dissolve 34.23 g of Sucrose (Sigma-Aldrich, Cat. No. S5390-1KG) in 70 mL of distilled water. Adjust the volume up to 100 mL and autoclave it.

**Note**: It is important to autoclave all the solutions except 0.8M Mannitol and 0.2M MES buffer for sterilization purposes.

**Solutions and Buffers:**

Respective solutions viz. 0.6 M Mannitol (Table S1), protoplast isolation buffer (Table S2), W5 buffer (Table S3), MMG buffer (Table S4), PEG-Calcium Transfection Buffer (Table S5), and 0.55 M sucrose solution (Table S6) were carefully prepared in aseptic condition.

**Table S1: 0.6 M Mannitol (Could be stored)**

| **Component** | **Source** | **Molecular weight** | **Stock concentration** | **Final concentration** | **Amount of Stock concentration** |
| --- | --- | --- | --- | --- | --- |
| D(-)Mannitol | Sigma  Cat. No. M1902-500G | 182.17 g/mol | 0.8 M | 0.6 M | 7.5 mL |
| Autoclaved  MiliQ H_2_O |  |  |  |  | 2.5 mL |
| Total |  |  |  |  | 10 mL |

**Table S2: Protoplast isolation buffer/Enzyme solution (Freshly prepared)**

| **Component** | **Source** | **Molecular weight** | **Stock concentration** | **Final concentration** | **Quantity from Stock** |
| --- | --- | --- | --- | --- | --- |
| D(-) Mannitol | Sigma  Cat. No. M1902-500G | 182.17 g/mol | 0.8 M | 0.6 M | 7.5 mL |
| MES (pH 5.7) | Sigma  Cat. No. RES0113M-A7 | 213.25 g/mol | 0.2 M | 10 mM | 0.5 mL |
| Cellulase R10 | Yakult, Japan  CELLULASE “ONOZUKA” R-10 | / | / | 1.5% | 0.15 g |
| Macerozyme R10 | Yakult, Japan  MACEROZYME® R-10 | / | / | 0.75% | 0.075 g |
| CaCl_2_.2H_2_O | Sigma  Cat. No. C3306-500G | 147.01 g/mol | 1 M | 1 mM | 0.01 mL |
| BSA | HIMEDIA  GRM3151 | / | 10% | 0.1% | 0.1 mL |
| *β*-mercaptoethanol | Sigma  444203 |  |  | 5 mM | 0.003 mL |
| Total |  |  |  |  | 10 mL |

Prepare the protoplast isolation buffer by sequentially adding the components listed in Table 2. Once MES, mannitol, cellulase, and macerozyme are added, incubate the mixture at 55°C for 10 minutes. Subsequently, cool the mixture to room temperature and add CaCl_2_.2H_2_O and BSA. Adjust the volume to 9 mL using sterile deionized water and maintain the pH at 5.8. Finally, bring the final volume to 10 mL and filter the solution using a 0.22 µm syringe filter. It is crucial to add β-mercaptoethanol after filtration.

**Critical:** Before adding CaCl_2_·2H_2_O and BSA, ensure that the enzyme-mannitol mixture is cooled down. Always add β-mercaptoethanol after filtration is done, as it can damage the filter.

**Table S3: W5 buffer, pH 5.8 (Could be stored at 4 ℃)**

| **Component** | **Source** | **Molecular weight** | **Stock concentration** | **Final concentration** | **Quantity from Stock** |
| --- | --- | --- | --- | --- | --- |
| MES (pH 5.7) | Sigma  Cat. No. RES0113M-A7 | 213.5 g/mol | 0.2 M | 1 mM | 0.5 mL |
| KCl | Sigma  Cat. No. P9541-500G | 74.55 g/mol | 2 M | 5 mM | 0.25 mL |
| NaCl | Sigma  Cat. No. 71376-1Kg | 58.44 g/mol | 5 M | 154 mM | 3.08 mL |
| CaCl_2_·2H_2_O | Sigma  Cat. No. C3306-500G | 147.01 g/mol | 1 M | 125 mM | 12.5 mL |
| D-(+)-Glucose | Sigma  Cat. No. G7021-1KG | 180.156 g/mol | 1 M | 1 mM | 0.1 mL |
| Total |  |  |  |  | 100 mL |

**Table S4: MMG buffer, pH 5.8 (Freshly prepared)**

| **Component** | **Source** | **Molecular weight** | **Stock concentration** | **Final concentration** | **Quantity from Stock** |
| --- | --- | --- | --- | --- | --- |
| D (-) Mannitol | Sigma  Cat. No. M1902-500G | 182.17 g/mol | 0.8 M | 0.6 M | 7.5 mL |
| MES (pH 5.7) | Sigma  Cat. No. RES0113M-A7 | 213.25 g/mol | 0.2 M | 4 mM | 0.2 mL |
| MgCl_2_ . 6H_2_O | Sigma  Cat. No. 63068-250G | 203.30 g/mol | 1 M | 15 mM | 0.15 mL |
| Total |  |  |  |  | 10 mL |

**Table S5: PEG-Calcium Transfection Buffer (Freshly prepared)**

| **Component** | **Source** | **Molecular weight** | **Stock concentration** | **Final concentration** | **Quantity from Stock** |
| --- | --- | --- | --- | --- | --- |
| PEG 4000 | Sigma  8.07490.1000 | / | / | 40 % (w/v) | 4 g |
| D(-)Mannitol | Sigma  Cat. No. M1902-500G | 182.17 g/mol | 0.8 M | 0.2 M | 7.5 mL |
| CaCl_2_·2H_2_O | Sigma  Cat. No. C3306-500G | 147.01 g/mol | 1 M | 100 mM | 1 mL |
| Total |  |  |  |  | 10 mL |

Prepare transfection buffer by adding the above-listed components and then mix it using an ultra sonicator at 45 ℃ temperature for 30 mins. Proper sonication is required to dissolve the PEG completely.

**Table S6: 0.55 M Sucrose solution (Freshly prepared)**

| **Component** | **Source** | **Molecular weight** | **Stock concentration** | **Final concentration** | **Quantity from Stock** |
| --- | --- | --- | --- | --- | --- |
| Sucrose | Sigma  Cat. No. S5390-1KG | 342.3 g/mol | 1M | 0.55 M | 5.5 mL |
| H_2_O |  |  |  |  | 4.5 mL |
| Total |  |  |  |  | 10 mL |

**Supplementary notes:**

**Note S1:** It is important to ensure that all cut strips are fully submerged in the protoplast isolation buffer. If a large number of cut strips is present, an additional amount of protoplast isolation buffer should be added. Check regularly to ensure the majority of the cut strips are submerged in the solution and not floating on the surface. Every 30 minutes, pause the vacuum and gently shake the flask/beaker with your hand for 30 seconds before resuming the vacuum.

**Note S2:** In case a cell strainer is not available, two layers of 37 µm nylon mesh can be used to filter protoplasts. Use 2-3 mL of W5 solution to prewet the nylon mesh. If the pore size is larger, using more layers is recommended.

**Note S3:** A vertical swing bucket rotor is preferred over a fixed-angle rotor. Add W5 solution through the tube wall, not directly into the pellet.

**Note S4:** To prepare cut tips, cut approximately 0.5 cm from the lower end of the tips and then expose them to a flame to blunt the ends. Be cautious to prevent the healthy protoplast interface from coming into contact with sucrose.

**Table S7: Primers used for this study**

| **Primer name** | **Sequence (5’-3’)** | **Purpose** |
| --- | --- | --- |
| 355-Gn1a-F | GTCCATCCACGCTGCTAGT | Screening primer for *OsCKX2* |
| 356-Gn1a-R | AGGGGTCGTCATTTTGAACG |  |
| 242-F1 | GGttcgaaATGGTGAGCAAGGGCGAG | GFP and BFP amplification primer |
| 243-R1 | gctctagagcTCACTTGTACAGCTCGTCCAT |  |
| 244-F2 | accaccctgTccCacggcgtgcagtgcttcag |  |
| 245-R2 | acgccgtGggAcagggtggtcacgagggtggg |  |
| 337-gR1-F | TAGGTCTCCCCACGTCGACCAgttttagagctagaa | *OsSD1* PTG cassette construction |
| 338-gR1-R | CGGGTCTCAGTGGGCGTGCTCtgcaccagccggg |  |
| 339-gR2-F | TAGGTCTCCCATGCCCGGAGCgttttagagctagaa |  |
| 340-gR2-R | CGGGTCTCACATGGCGGGTAGtgcaccagccggg |  |
| 373-gR1-F | TAGGTCTCCAGCCCCGCGGCGgttttagagctagaa | *OsCKX2*  PTG cassette construction |
| 374-gR1-R | CGGGTCTCAGGCTCTGGGACGtgcaccagccggg |  |
| 375-gR2-F | TAGGTCTCCCGGGGGAGGAACgttttagagctagaa |  |
| 376-gR2-R | CGGGTCTCACCCGCTCCGGCGtgcaccagccggg |  |
| 377-gR3-F | TAGGTCTCCCACCCCCGCCATgttttagagctagaa |  |
| 378-gR3-R | CGGGTCTCAGGTGCGGCTGAGtgcaccagccggg |  |
| 838-GAT-gRNA-1-F | TAGGTCTCCAGGCATATGTCTgttttagagctagaa | *AtGAT*  PTG cassette construction |
| 839-GAT-gRNA-1-R | CGGGTCTCAGCCTAGAAATGGtgcaccagccggg |  |
| 840-GAT-gRNA-2-F | TAGGTCTCCTCCAACTATTCCgttttagagctagaa |  |
| 841-GAT-gRNA-2-R | CGGGTCTCATGGACGCTTTGAtgcaccagccggg |  |
| 426-gRC1-F | TAGGTCTCCATTTGCTTCATGgttttagagctagaa | *CaLCY*  PTG cassette construction |
| 427-gRC1-R | CGGGTCTCAAAATGCCGACTGtgcaccagccggg |  |
| 428-gRC2-F | TAGGTCTCCGTATGAGGTTGGgttttagagctagaa |  |
| 429-gRC2-R | CGGGTCTCAATACTGCAATAGtgcaccagccggg |  |
| 408-gRC1-F | TAGGTCTCCTGGGCAAGCTCGgttttagagctagaa | *CaM41*  PTG cassette construction |
| 409-gRC1-R | CGGGTCTCACCCACGTGGAACtgcaccagccggg |  |
| 410-gRC2-F | TAGGTCTCCGAGCTCTCCTATgttttagagctagaa |  |
| 411-gRC2-R | CGGGTCTCAGCTCTGAATTCGtgcaccagccggg |  |
| 414-CaM41-F | AGAATATGTATGTTGCCGTCATG | Screening primer for *CaM41* |
| 415-CaM41-R | AGGGAGTTGAAGAAGGTACCA |  |
| 430-Ca_LCY-F | AGCATGTTTGGAAGGATACC | Screening primer for *CaLCY* |
| 431-Ca_LCY-R | AGTAGAAAAGGGAATGGCTGC |  |
| 610-AtGATase-Sc-F | TGCACCCGCCATGGATGTACTT | Screening primer for *AtGAT* |
| 911-AtGAT-R | GGAATGGCCCTGTACGAATG |  |
| 618-F1 | ccaagcttCTTTTTTTCTTCTTCTTCGTTCATACAG | For AtU6-26 cloning to replace OsU3 |
| 846-AtU6-R1 | cctgcaggaaaaccgagacctcggtctcctgccATCACTACTTCGACTCTAGC |  |
| 621-E-CaMV35s-F | GCCTGCAGGTCCACAATGAGACTTTTCAACAAAGGG | For E-CaMV35S cloning to replace *OsUbi10* |
| 622-E-CamV35s-R | gcttcgaagcctgcttttttgtacaaacttgttcagcgtgtcctctcc |  |
| 623-AtUBI10-F | cctgcaGGTCCACAAGATCAGGATATTCTTGTTTAAGATG | For AtUbi10 cloning to replace OsUbi10 |
| 748-R1-AtUbi10 | cttcgaaCGATCTAAGATTAACAGAATC |  |
| Gr1F | TAGGTCTCCCATGTCAGCAGCgttttagagctagaa | *OsSWEET14*  PTG cassette construction |
| Gr1R | CGGGTCTCACATGCATGCCCTtgcaccagccggg |  |
| Gr2F | TAGGTCTCCCCCCCTCCAACCgttttagagctagaa |  |
| Gr2R | CGGGTCTCAGGGGTTTATATAtgcaccagccggg |  |
| 201-Sweet14-P-F | TCCCATGCATTGAGGACAGA | Screening primers for *OsSWEET 14* |
| 202-Sweet14-P-R | CCAGGGATGCTGAAGAGACA |  |

**Supplementary figures**

**
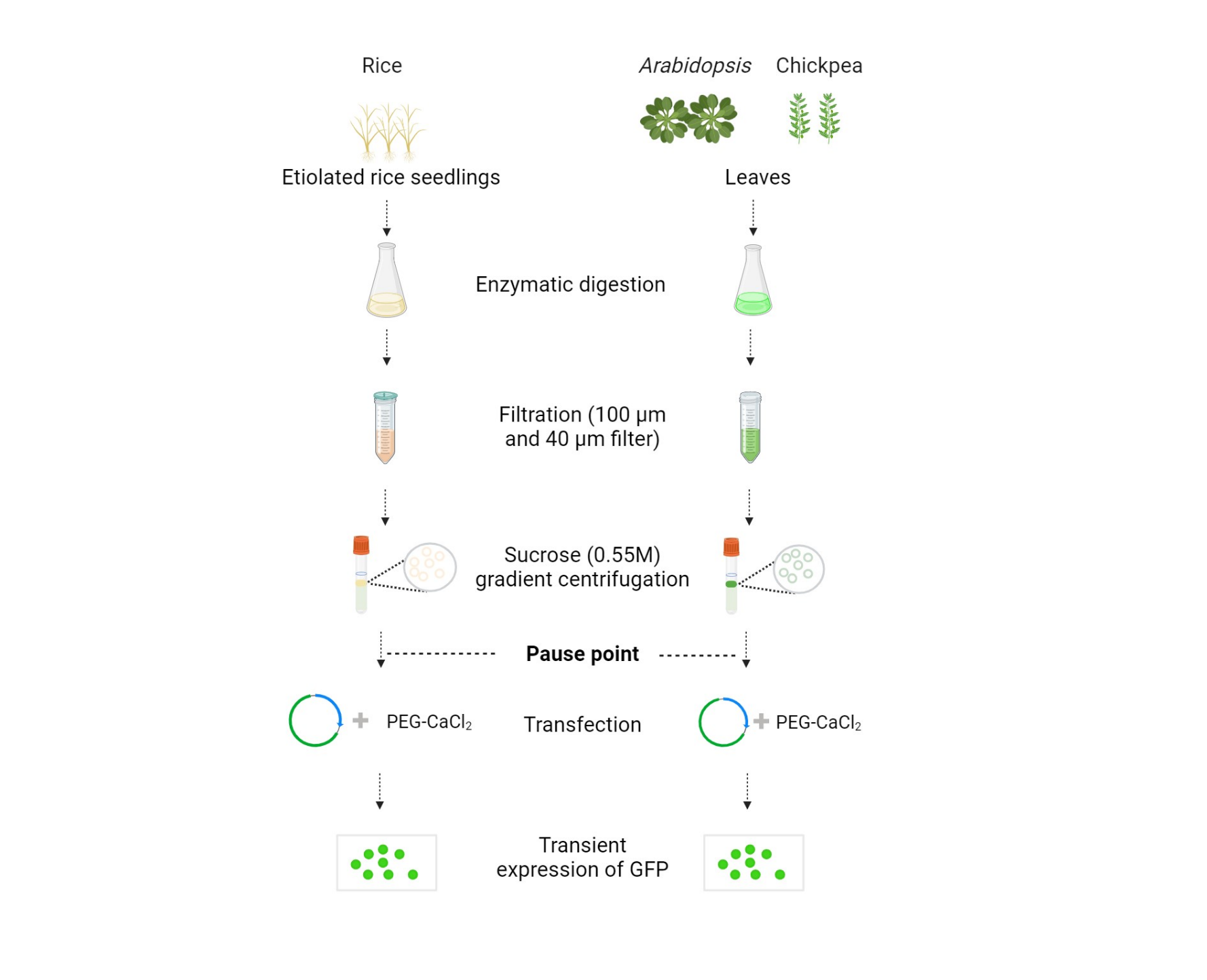
**

Fig S1: Schematic representation of protoplast isolation and transfection procedure


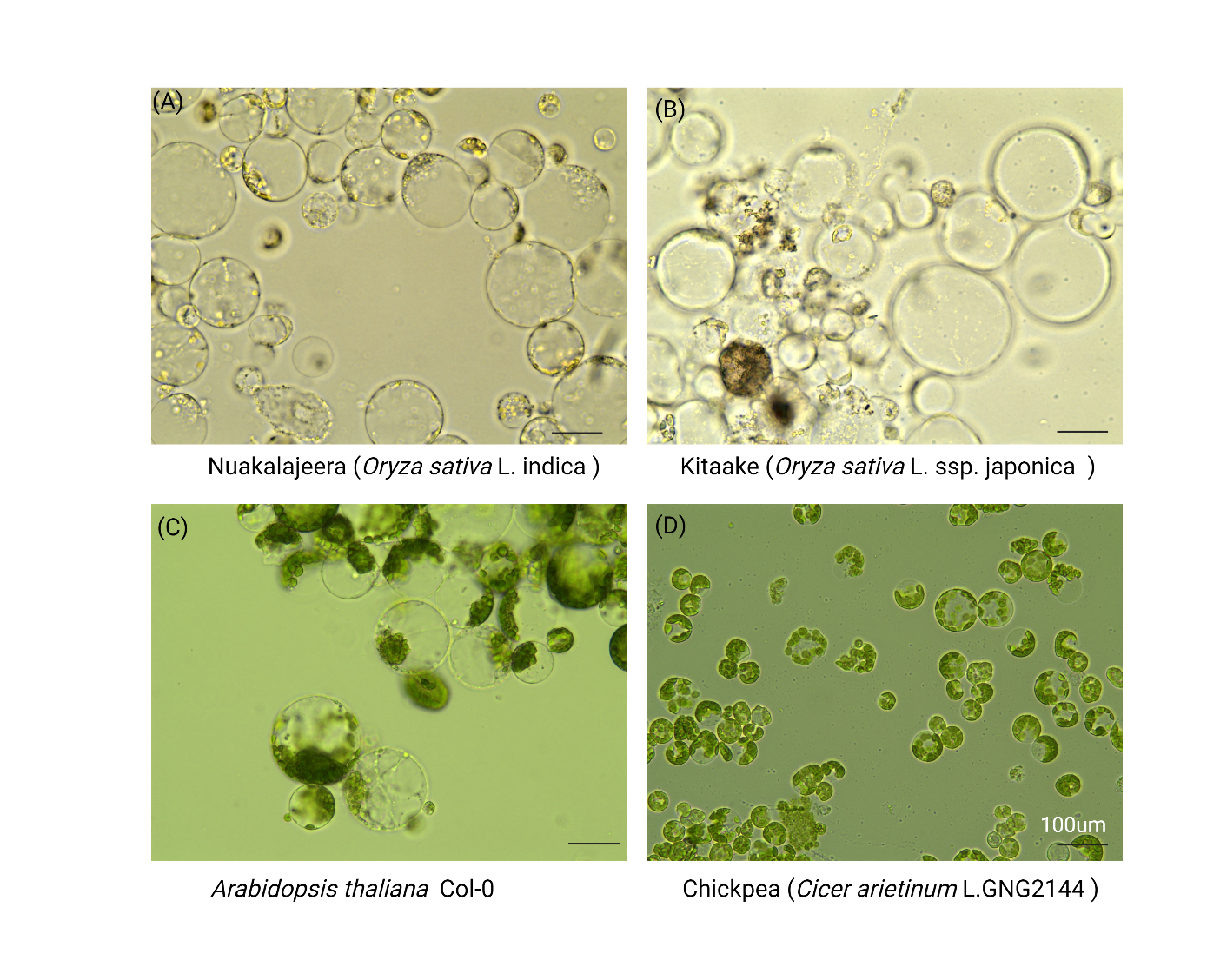


Fig S2: Morphology of monocot and dicot protoplasts at 40X bright field. **A** and **B** images showing etiolated protoplasts of rice; Nuakalajeera (*Oryza sativa* L. indica) and Kitaake(*Oryza sativa* L. subsp. Japonica) respectively. **C** and **D** images showing green protoplasts of Arabidopsis (*Arabidopsis thaliana* Col-0), and chickpea (*Cicer arietinum* L. GNG2144) respectively.


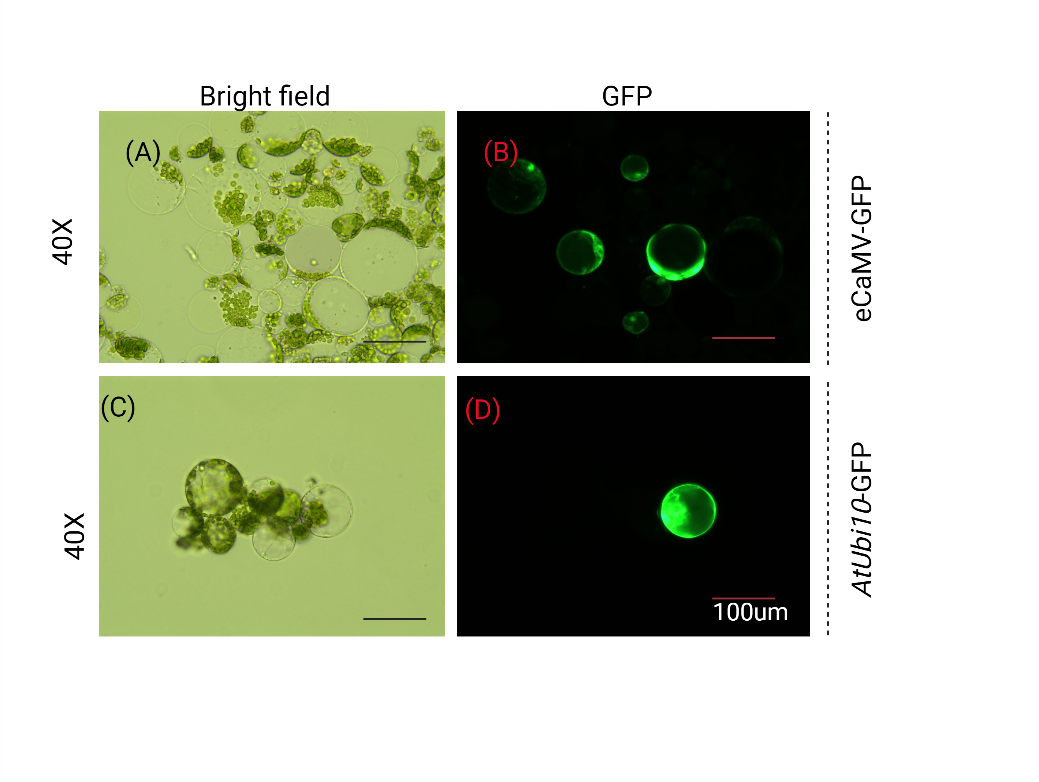


Fig S3: Transfection efficiency study in *Arabidopsis* using different promoters. Images showing transfected protoplasts isolated using *eCaMV* promoter for the expression of GFP gene viewed under **A** bright field and **B** GFP filter. Images showing transfected protoplast isolated using *AtUbi10* promoter for the expression of GFP gene viewed under **C** bright field and **D** GFP filter.


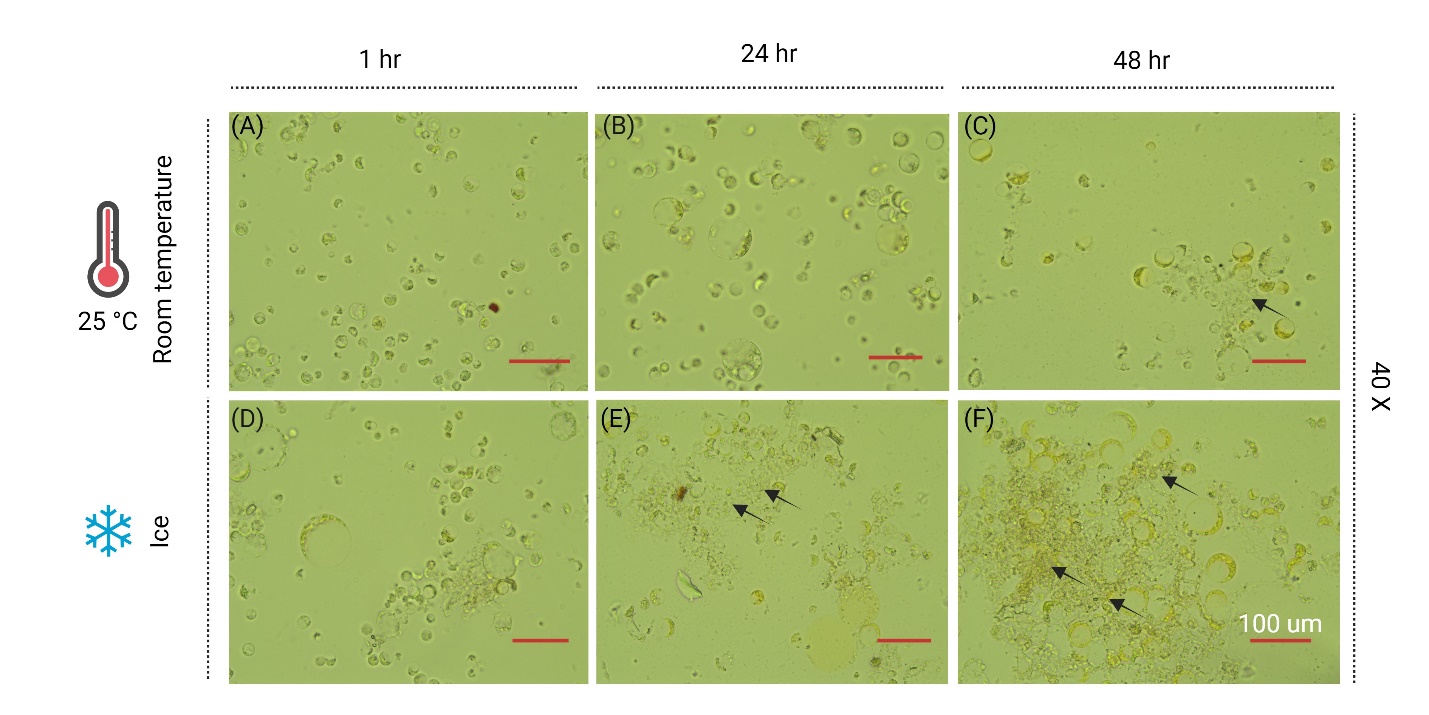


Fig S4: Optimizing the storage of protoplasts at different conditions. **A-D**, Bright-field images of protoplasts stored at room temperature and on ice after 1 hr. **B-E**, after 24 hr. **C-F** after 48 hr. Black arrows highlight clusters of ruptured protoplasts.


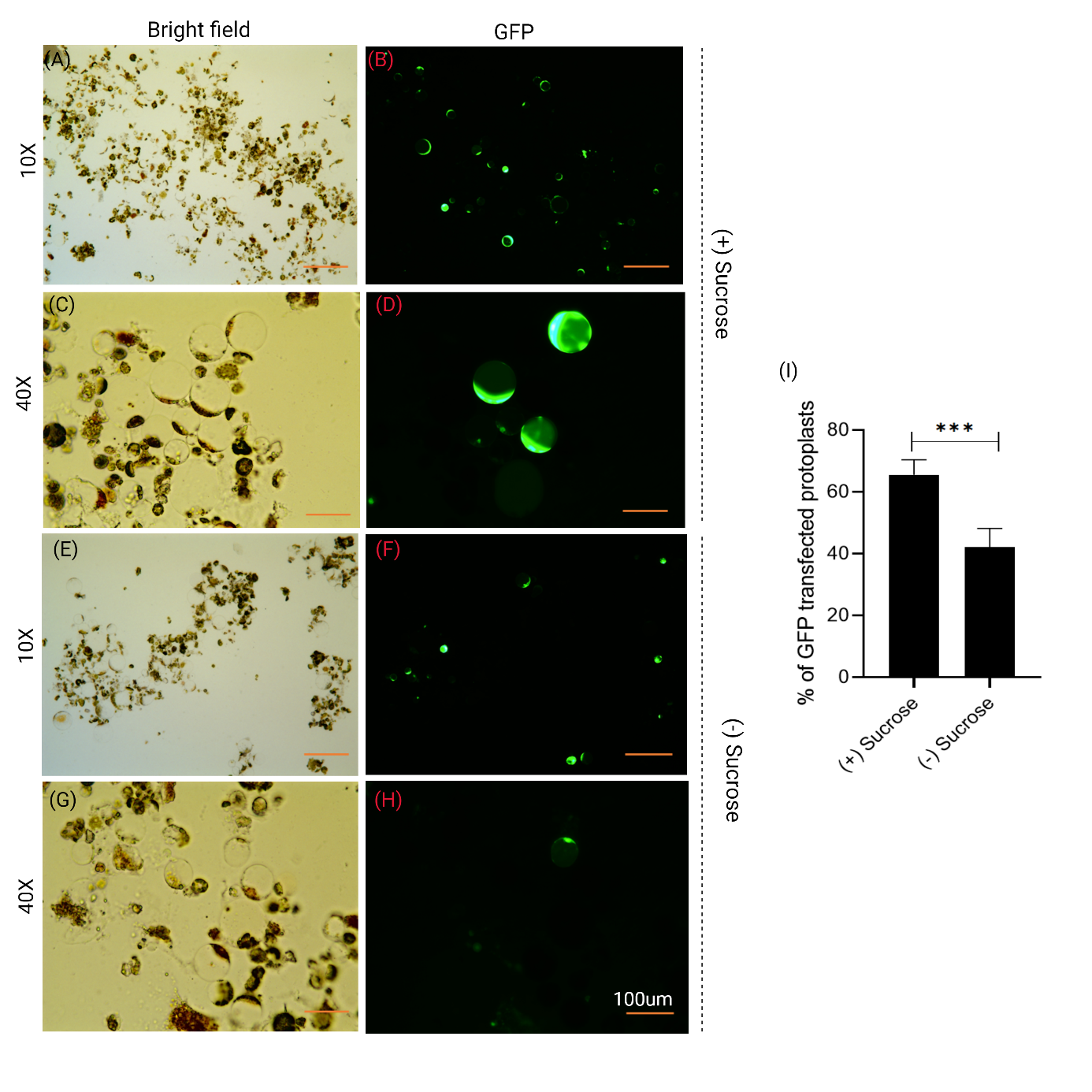


Fig S5: Optimization of transfection efficiency of *Arabidopsis* protoplasts using (+) sucrose gradient and (-) sucrose gradient. **A-D** Bright field and GFP filter image of protoplasts isolated with (+) sucrose gradient. **E-H** Bright field and GFP filter image of protoplasts isolated without (-) sucrose gradient. **I** Represents the transfection efficiency of GFP transfected protoplasts with and without sucrose gradient. Each bar represents mean ± SE for three replicates.


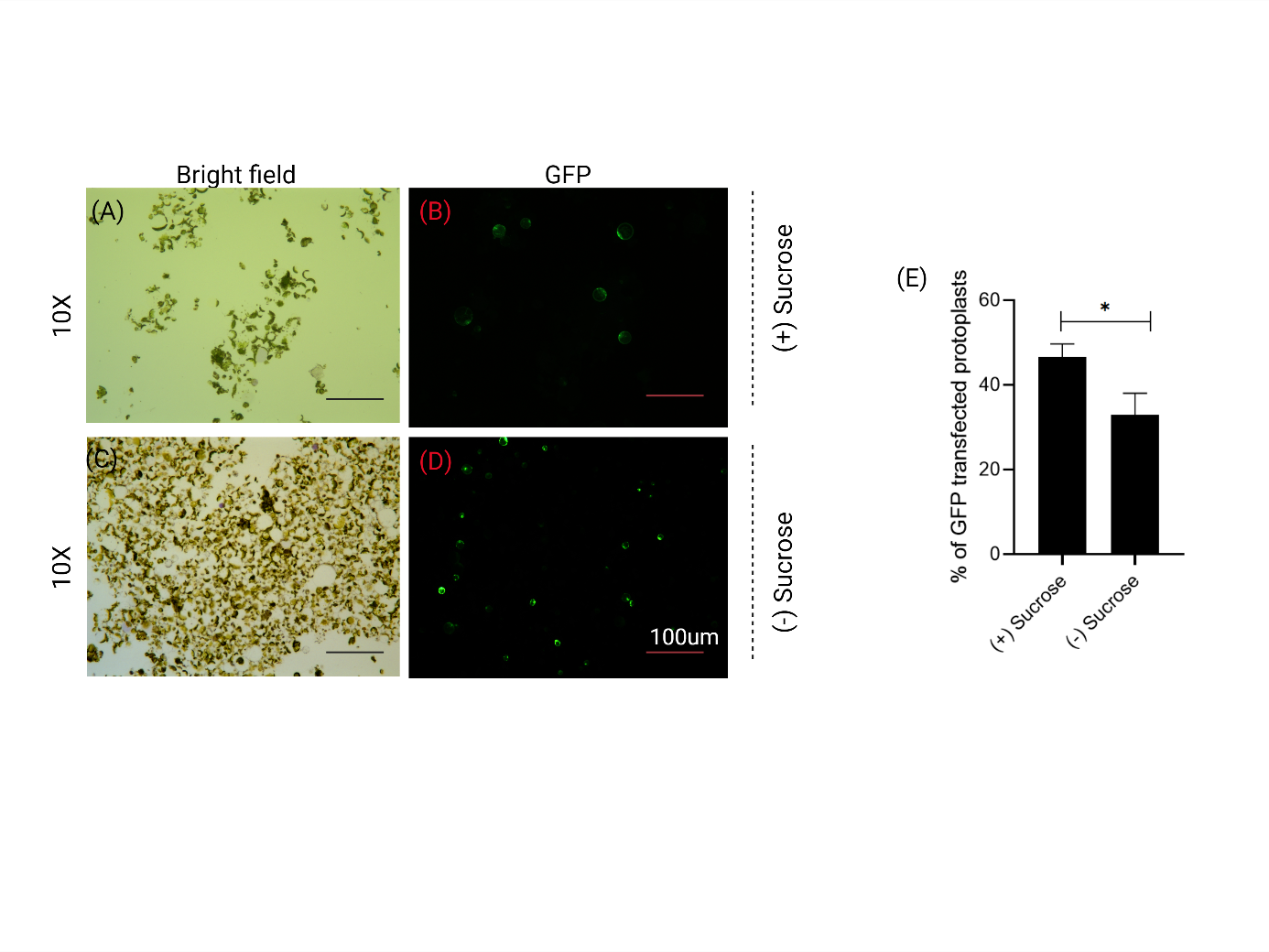


Fig S6: Optimization of transfection efficiency of *chickpea* protoplasts using (+) sucrose gradient and (-) sucrose gradient. **A** and **B** Bright field and GFP filter image of protoplasts isolated with (+) sucrose gradient. **C** and **D** Bright field and GFP filter image of protoplasts isolated without (-) sucrose gradient. **E** Represents the transfection efficiency of GFP transfected protoplasts with and without sucrose gradient. Each bar represents mean ± SE for three replicates.

**Troubleshooting**

1. **Protoplasts have low viability after isolation**. When working with different varieties like Nuakalajeera (Indica) and Kitaake (Japonica), or different plant species, varietal differences can pose challenges. This requires adjustments in enzyme concentration during digestion, incubation time, and the age of the tissue. We used 10-12-day-old etiolated rice seedlings and 2-3-week-old green leaves for Arabidopsis and chickpea as explants for protoplast isolation. Cutting thin tissues (0.5mm-1mm) is crucial for efficient protoplast digestion.
2. **Protoplasts shrinkage and rupturing**. Protoplasts shrinkage can occur if solutions are not carefully prepared. Variations in reagent concentrations and buffer pH can lead to protoplast shrinkage. Therefore, ensure that all solutions and buffers, especially MMG, are meticulously prepared with the correct amounts. To prevent protoplast rupturing, never exceed a maximum shaking speed of 80 rpm during the digestion process. Pre-wet the cell strainers with an appropriate amount of W5 buffer to facilitate the filtration process.
3. **Poor health of protoplasts**. Prepare 0.55M sucrose carefully with the correct amount. After adding sucrose to the protoplasts, mix it thoroughly before adding W5 solution.
4. **Low transfection efficiency**. The concentration and purity of plasmids are critical for transfection efficiency. Always use transfection-grade plasmids isolated using an appropriate kit, and quantify the plasmids using Qubit or agarose gel. Since calf serum is used in the transfection process, there is a higher risk of fungal contamination. To prevent fungal contamination, perform all steps meticulously in aseptic conditions.
